# PhageScout: Protease Cleavage Site Prediction Using an Experimental Substrate Phage Display Motif-Based Approach

**DOI:** 10.3390/ijms27177593

**Published:** 2026-08-25

**Authors:** Enoch Yu, Matthew L. Holding, Rex Huang, Andrew Chan, Cherie Teney, Colin A. Kretz

**Affiliations:** 1 Department of Medicine, Thrombosis and Atherosclerosis Research Institute, McMaster University, Hamilton, ON L8L 2X2, Canada; 20ey3@queensu.ca (E.Y.);; 2 Life Sciences Institute, University of Michigan, Ann Arbor, MI 48109, USA; 3 Department of Ecology and Evolutionary Biology, University of Michigan, Ann Arbor, MI 48109, USA; 4 Department of Biochemistry and Biomedical Sciences, McMaster University, Hamilton, ON L8S 4K1, Canada; 5 Hamilton Health Sciences, Hamilton, ON L8N 3Z5, Canada

**Keywords:** proteases, proteomics, enzyme specificity, computational biology

## Abstract

Identification of protease cleavage sites is essential for understanding biological regulation and disease mechanisms, yet many predictive approaches rely on annotated substrates and curated databases, limiting performance for poorly characterized proteases. We present PhageScout, a framework for database-independent generation of protease-specific features to predict cleavage sites using de novo experimental substrate phage display screening. We screened a randomized 5-mer phage display library against two neutrophil serine proteases (cathepsin G, elastase). Cleaved peptides generated position weight matrices (PWMs) and peptide enrichment scores to evaluate cleavage-site likelihood across substrate sequences. Sequence-derived scores were integrated with structural features, including accessibility and flexibility, using XGBoost classification models. Performance was benchmarked against annotated cleavage sites from the MEROPS peptidase database as reference data. Phage-derived PWM scores alone captured protease preferences and discriminated cleavage sites from background sites. Without model fitting, PWM scores achieved an area under the curve (AUC) of 0.756 (95%CI: 0.714–0.797) (cathepsin G) and 0.787 (95%CI: 0.753–0.821) (elastase). Combining broad and specific phage-derived scores improved cathepsin G prediction (AUC = 0.783), whereas this improvement was not observed for elastase. Compared to only phage-derived features, XGBoost models integrating phage sequence and structural features provided modest gains for elastase (AUC = 0.775 to 0.806), with phage-derived features ranking among the strongest predictors, but not cathepsin G (AUC = 0.702 to 0.710). Our findings demonstrate that PhageScout can use experimentally derived cleavage signatures to generate protease-specific predictive features and prioritize protease cleavage sites, providing a framework that warrants further validation across diverse proteases and biological contexts.

## 1. Introduction

Proteases are essential enzymes that catalyze the hydrolysis of peptide bonds, serving as critical regulators of protein function and cellular signaling. Proteases govern diverse physiological roles from digestion and blood coagulation to immune responses [1]. However, the same catalytic process to maintain homeostasis can drive disease when dysregulated, such as in tumor invasion and metastasis, extracellular matrix remodeling in cardiovascular diseases, or inflammatory diseases [2]. Consequently, characterizing protease–substrate interactions and cleavage sites is critical for both understanding fundamental biological mechanisms and identifying therapeutic targets [1,3,4].

Predicting cleavage sites among substrates remains a major challenge. Experimental screening of individual cleavage events in proteins is traditionally labor-intensive and low-throughput. To address this, the field has largely turned toward database-reliant computational methods that use known protease substrates, prior screens, or expert knowledge to predict cleavage in new sequences. Models like PROSPER [5], CasCleave [6], and IProt-Sub [7] draw from established repositories like MEROPS, CutDB [8], CASBAH [9], and PMAP [10], integrating substrate primary sequence data with structural and physicochemical features. Protein language models (PLMs), often trained on UniProt protein data, aim to understand protein folding and context, with PLM frameworks like MP-BERT [11] and ESM-2 [12] fine-tuned on MEROPS datasets to predict cleavage sites, as applied in MPCutter [13] or Cifuentes et al. [14]. Similar machine learning-based approaches include DeepCleave [15] and Unizyme [16], which apply structural feature learning onto protease–substrate pairs from the MEROPS database.

Our approach seeks to supplement these existing efforts by addressing a key limitation: most current models are reliant on inference from known substrates and prior substrate screens. This may create a literature imbalance that favors well-studied enzymes and limits characterization of proteases in complex environments, including those containing inhibitors or modulators, along with mutant variants and new proteases. However, small-peptide substrate phage display, which displays millions of randomized short peptides on bacteriophage, is well suited for de novo protease profiling because it directly measures sequence cleavage site preferences without requiring prior knowledge of endogenous substrates [17,18]. This enables the generation of protease-specific substrate profiles that can be used to predict cleavage site preferences across diverse protein cut sites and biological contexts [18,19].

Herein, we introduce PhageScout, a method that integrates de novo experimental substrate profiling data to predict P1-P1’ cleavage sites within substrates. The principal advance of PhageScout is protein-wide cleavage-site scoring using experimentally derived, protease- or setting-specific sequence preferences, demonstrating the utility of substrate phage display for cleavage-site prediction without requiring curated annotations or prior substrate knowledge for feature generation. Our method offers a cost-effective and high-throughput solution for prioritizing cut sites that may help overcome constraints of datasets and better reflecting protease activity in non-standard or complex physiological conditions, as a standalone tool or supplemental feature to augment existing tools or feature sets. To demonstrate the utility of this de novo approach, we apply our screening platform to neutrophil serine proteases cathepsin G and neutrophil elastase, which have distinct yet partially overlapping cleavage specificities along with critical roles in normal innate immune defense or disease pathogenesis for conditions like emphysema, stroke, or rheumatoid arthritis [20,21,22]. As such, these two proteases provide a useful test case for evaluating protease-specific cleavage-site discrimination, with subsequent performance validation conducted on MEROPS gold-standard established substrates.

## 2. Results

### 2.1. Characterization of Phage-Derived Protease Cleavage Site Preferences

**Figure 1:**
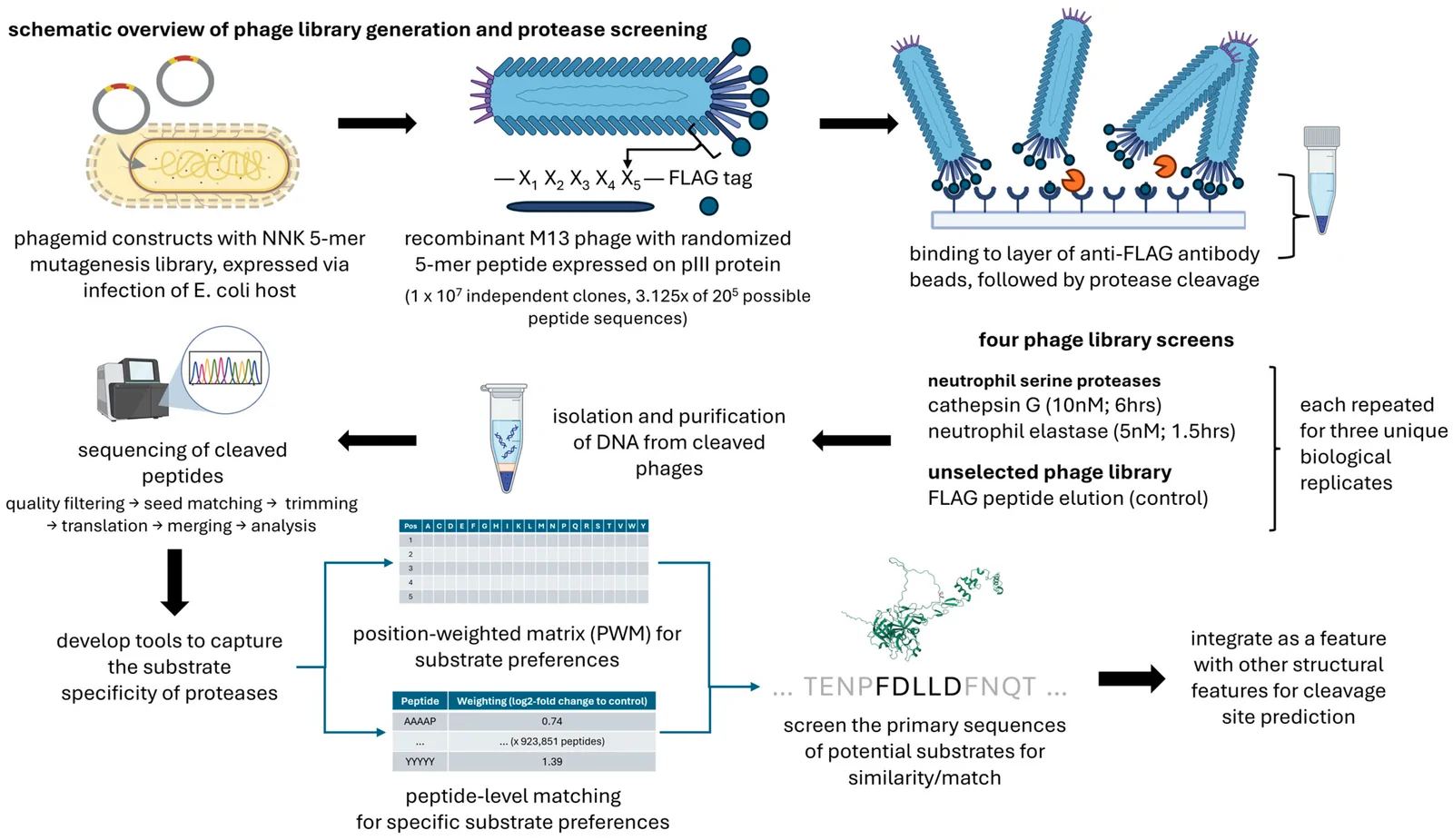
Experimental workflow for high-throughput substrate phage display protease profiling and generation of the randomized 5-mer phage library. pIII: M13 bacteriophage filamentous phage protein III.

High-throughput sequencing identified an average of 0.95 million unique cleaved peptides recovered from the phage screen, with a mean and median peptide count of 16.7 and 2, respectively (Figure 1). This corresponded to approximately 29.7% coverage of 20^5^ possible 5-mers (representing 20 possible amino acids per position) following removal of peptides with stop codons. After filtering low-count peptides, approximately 0.92 million unique cleaved peptides were detected. Totals of 9577 and 8364 peptides were identified as significantly cleaved (log2-fold change > 1; Bonferroni-corrected *p*-value < 0.001) by cathepsin G and elastase, respectively (Table S2). Log2-fold enrichment values followed a normal distribution for all proteases (Figure S2).

**Figure 2:**
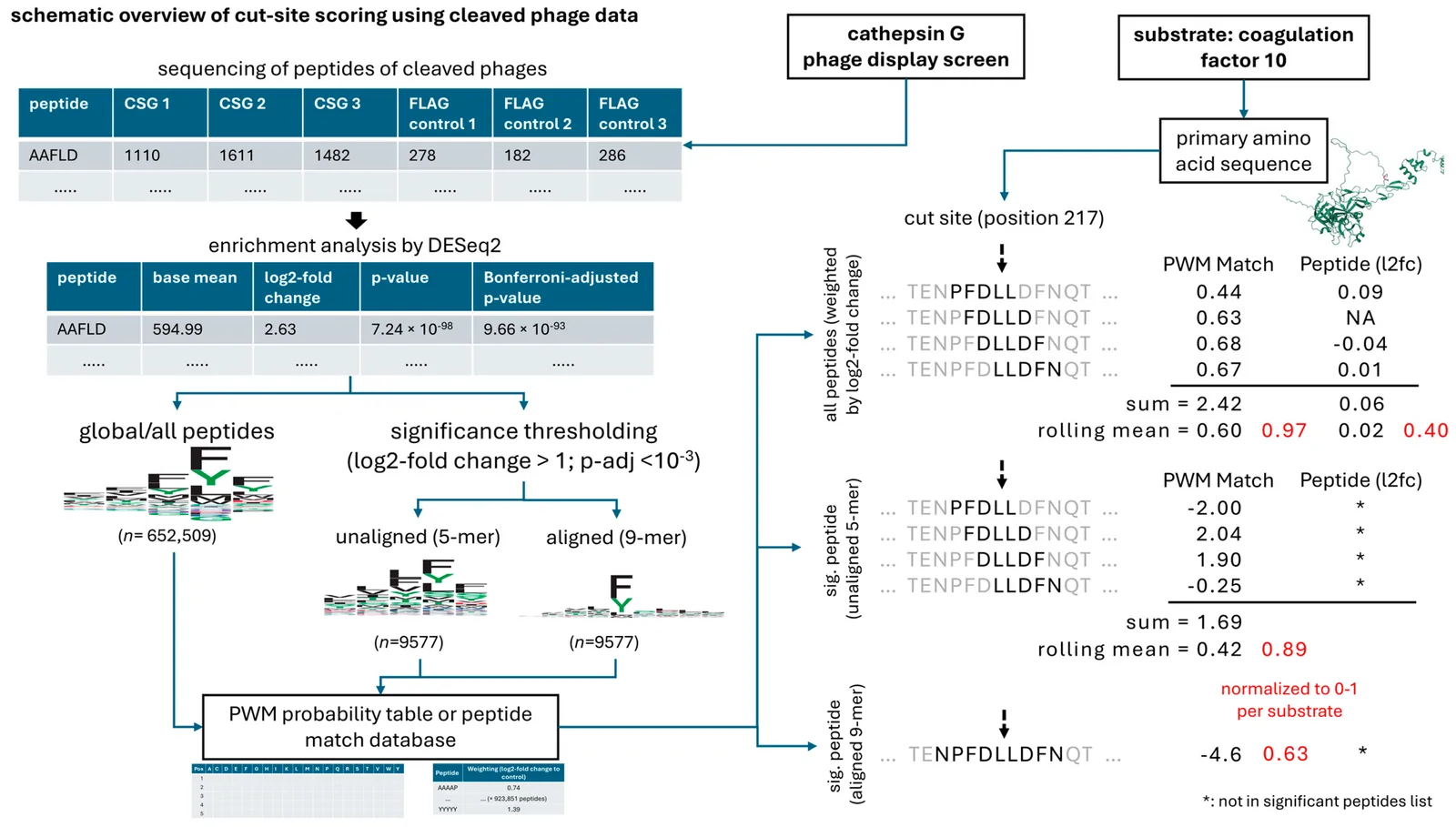
Workflow for protease cut site score calculations, involving either position weighted matrix (PWM) scoring or peptide profile-based scoring, which matches the cut site to amino acid probabilities or specific sequences of cleaved peptides, respectively. PWMs and peptide profiles were generated after DESeq2 analysis, which evaluated each peptide’s enrichment relative to the unselected library control. *p*-adj: Bonferroni-corrected adjusted *p*-value; l2fc: log2-fold change in peptide enrichment relative to control.

**Figure 3:**
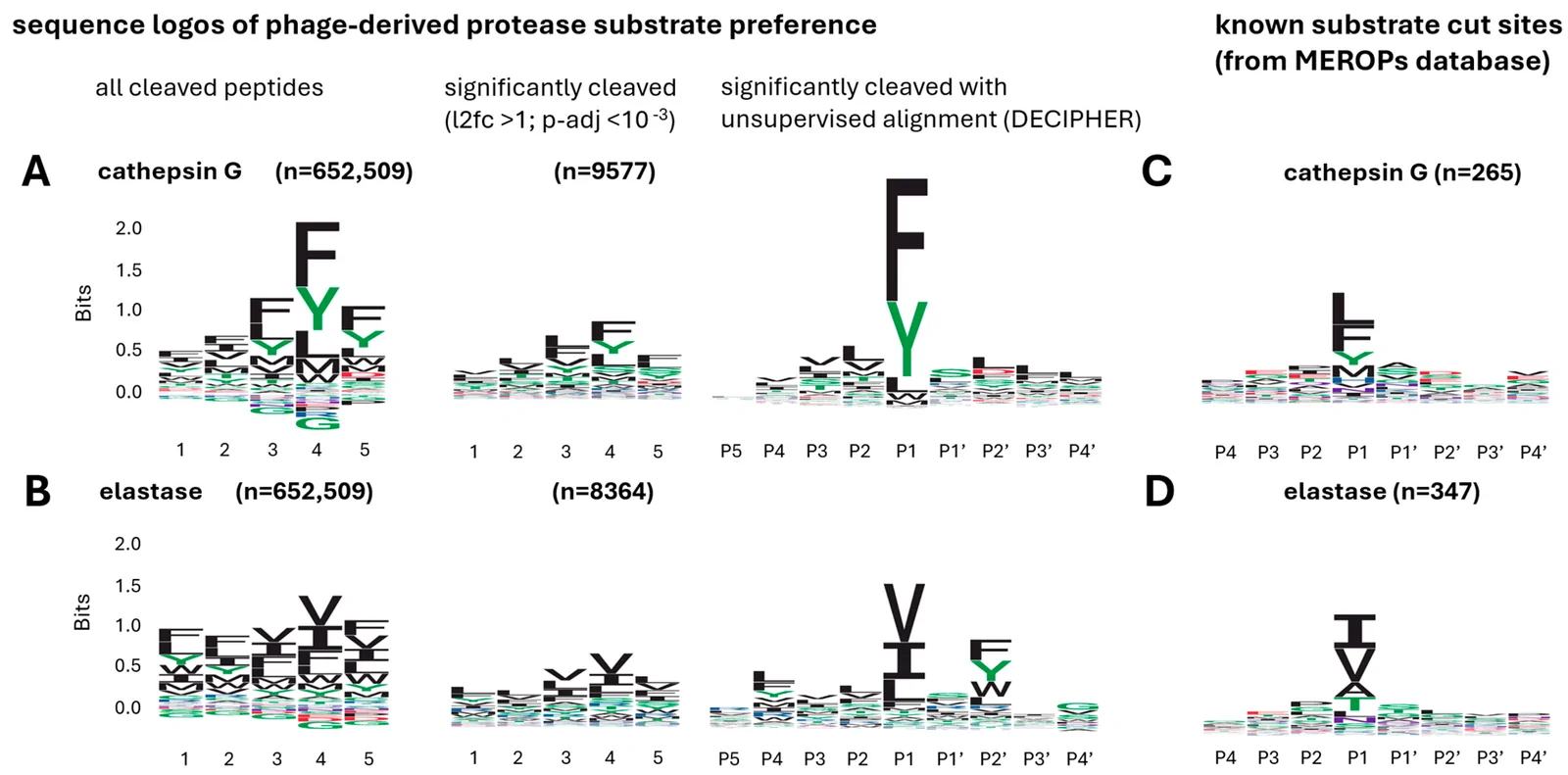
Characterization of cathepsin G and neutrophil elastase cleavage motifs. (**A**,**B**) Position weight matrices of significantly cleaved peptides, relative to the unselected library control, determined by DESeq2 (**left**). Sequences underwent unsupervised alignment by DECIPHER into 9-mer space (**right**). (**C**,**D**) Sequence logos of known substrate cut sites for each protease from the MEROPS peptidase database. All letters represent amino acids.

Position weight matrices (PWMs) derived from significantly enriched peptides revealed clear substrate preferences for cathepsin G and elastase. Cathepsin G favored large hydrophobic residues phenylalanine (F) and tyrosine (Y), whereas elastase showed a more restricted preference for smaller aliphatic residues valine (V), isoleucine (I), and leucine (L) (Figure 2, Figure 3A and Figure S3). Following unsupervised alignment by DECIPHER, dominant P1 motifs for F/Y/L/M (cathepsin G) and V/I/L (elastase) emerged, along with subsite P2 preferences such as serine for cathepsin G and small hydrophobic residues for elastase. Motifs were similar to those identified in known MEROPS cleavage sites (Figure 3B and Figure S1).

### 2.2. Phage-Derived Sequence Scores Capture Differences Between Known Cleavage Sites and Background Unannotated Sites

**Figure 4:**
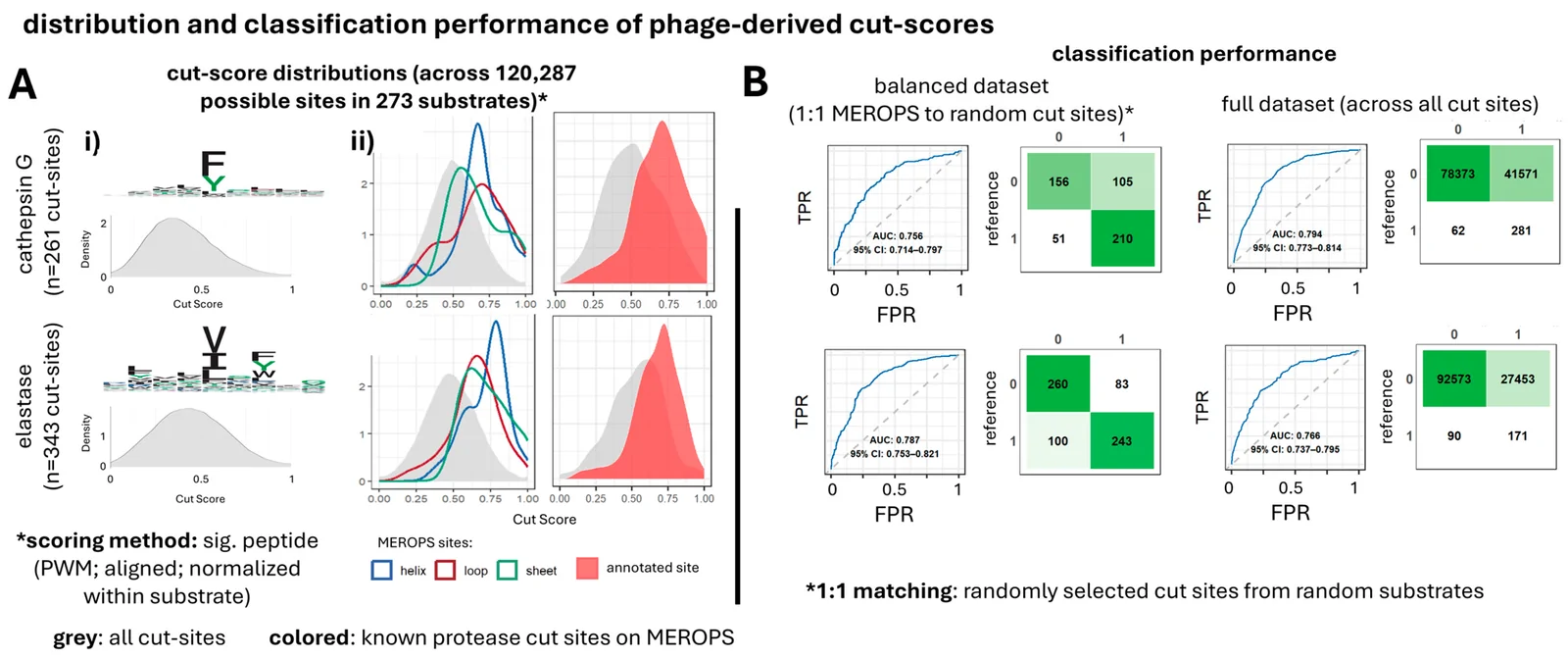
Evaluation and distributions of phage-derived cut scores, using the aligned PWM from significantly cleaved phage peptides. (**A**) Distributions of cut scores calculated across all possible cut sites in 273 substrates. (**i**) Density distribution of all cut scores. (**ii**) Density plots of all cut scores overlaid with cut scores of known MEROPS cleavage sites, subgrouped by secondary structure type. (**B**) Performance of cut scores in classifying cleavage and non-cleavage across substrate sites, evaluated by ROCs and confusion matrices with a Youden’s threshold. Cut scores were evaluated in a balanced 1:1 MEROPS to control setting or in a full dataset across all cut sites.

To evaluate the scoring methods, we defined a representative sub-proteome by pooling the 273 substrates across the two enzymes, yielding 120,287 potential cut sites. Cut site scoring using phage-derived PWMs produced approximately normally distributed cut scores across the sub-proteome (Figure 4A and Figure S4). Known cleavage sites from curated MEROPS substrates were used to benchmark our phage-derived features. These established cleaved sites regularly scored higher than background sites within the same proteins (Figure S5). For example, annotated cleavage sites in coagulation factor X (cathepsin G) and antithrombin III (elastase) ranked among the highest-scoring positions within their respective proteins (Figure S5).

Across substrates, known cleavage sites exhibited a clear rightward shift in cut score distributions relative to all possible sites, indicating discrimination by phage-derived sequence features to distinguish cleaved sites from background unannotated sites. This trend was observed for both physiological and non-physiological substrates (Figure 4B and Figure S6) and across all secondary structure contexts of cut sites, although cleavage sites within α-helical regions tended to show elevated scores than sites at loops or sheets (Figure 4B and Figure S7).

To assess whether these motifs represented meaningful biochemical signatures rather than random sequence patterns, PWMs derived from enriched peptides were compared against 10,000 randomly generated PWMs. For cathepsin G and elastase, experimentally derived PWMs ranked from the 95th to 100th percentile of all PWMs, confirming that observed motifs routinely capture biological cleavage site preferences (Figure S8).

### 2.3. Phage-Derived Scores Predict Cleavage Sites in Both Balanced and Global Cut Site Contexts

To evaluate the baseline predictive power of phage-derived sequence scores, we evaluated their performance on discriminating known cleavage sites against random controls in a balanced 1:1 dataset. Cut scores alone, without any tuning or fitting, demonstrated moderately strong discriminative power. PWM scores from significant peptides with unsupervised alignment yielded AUC values of 0.756 (95%CI: 0.714–0.797) for cathepsin G and 0.787 (95%CI: 0.753–0.821) for elastase (Figure 4B), with precision-recall AUPRCs of 0.753 (95%CI: 0.705–0.802) and 0.723 (95%CI: 0.667–0.774) respectively (Figure S13). Performance for cathepsin G was consistent across alternative PWM scoring methods (AUC 0.706–0.756; AUPRC 0.657–0.753), but improved further (AUC 0.783, 95%CI: 0.744–0.822; AUPRC 0.784, 95%CI: 0.737–0.833) when combining scores from the aligned significant peptide PWM with a broader PWM generated from the full peptide population (Figure S9). This improvement, though non-significant, suggests that integrating both dominant sequence motifs and broader permissive sequence features may potentially enhance cleavage prediction. Elastase exhibited more variable performance across PWM scoring methods (AUC 0.654–0.803), with the highest from using unnormalized PWM scores from aligned significant peptides (AUC 0.803; AUPRC: 0.764), and did not show a clear benefit from inclusion of broader sequence signatures (AUC 0.741; AUPRC: 0.723) (Figure S10).

Following the selection of a score threshold using Youden’s method, cathepsin G yielded the highest sensitivity (0.858) and Matthews Correlation Coefficient (MCC) (0.462) with the combination of dominant and broader features (Table S3), suggesting moderate predictive power. Specificity and PPV were comparatively low at 0.586 and 0.675, respectively. Elastase showed the strongest performance with dominant features alone, with the highest MCC at 0.499 (Table S3). Unlike cathepsin G, sensitivity and specificity for elastase were similar, at 0.764 and 0.735, respectively.

Subsequently, we evaluated the performance of scores to distinguish known cleavage sites against all cut sites of the 273 substrates tested. In this broad context, PWM scores across different methods yielded comparable performance, with AUC 0.703–0.768 for cathepsin G (Figure S11) and AUC 0.662–0.804 for elastase (Figure S12). Similar to the balanced dataset, cathepsin G performance increased when integrating specific and broad PWM scoring methods (AUC 0.780; 95%CI: 0.752–0.808), which did not represent a significant difference (Figure S11), but not for elastase (AUC 0.749; 95%CI: 0.725–0.772) (Figure S12). Due to the strong class imbalance, AUPRCs remained low, but with a 2- to 5-fold increase over baseline prevalence for both cathepsin G (baseline 0.221%; AUPRCs 0.004–0.01) and elastase (baseline 0.285%; AUPRCs 0.005–0.011), indicating weak-to-moderate discrimination of cleaved sites from background.

When using a selected threshold, the MCC across all methods remained similarly low, around 0.03 to 0.06 (Table S3). Each scoring method exhibited a different tradeoff of sensitivity and specificity. For cathepsin G, the broad PWM score based on all peptides and any method combining this score tended toward high sensitivity, while the other methods tended toward higher specificity. The highest PPV was 0.6% (95%CI: 0.5–0.7%), in the aligned PWM score. As for elastase, all PWM scores tended toward high sensitivity, except when broad and specific scoring methods were combined, which shifted toward higher specificity. The highest PPV was 0.8% (95%CI: 0.7–0.9%), also in the aligned PWM score, while NPV remained near 99.9% for both proteases across methods.

**Figure 5:**
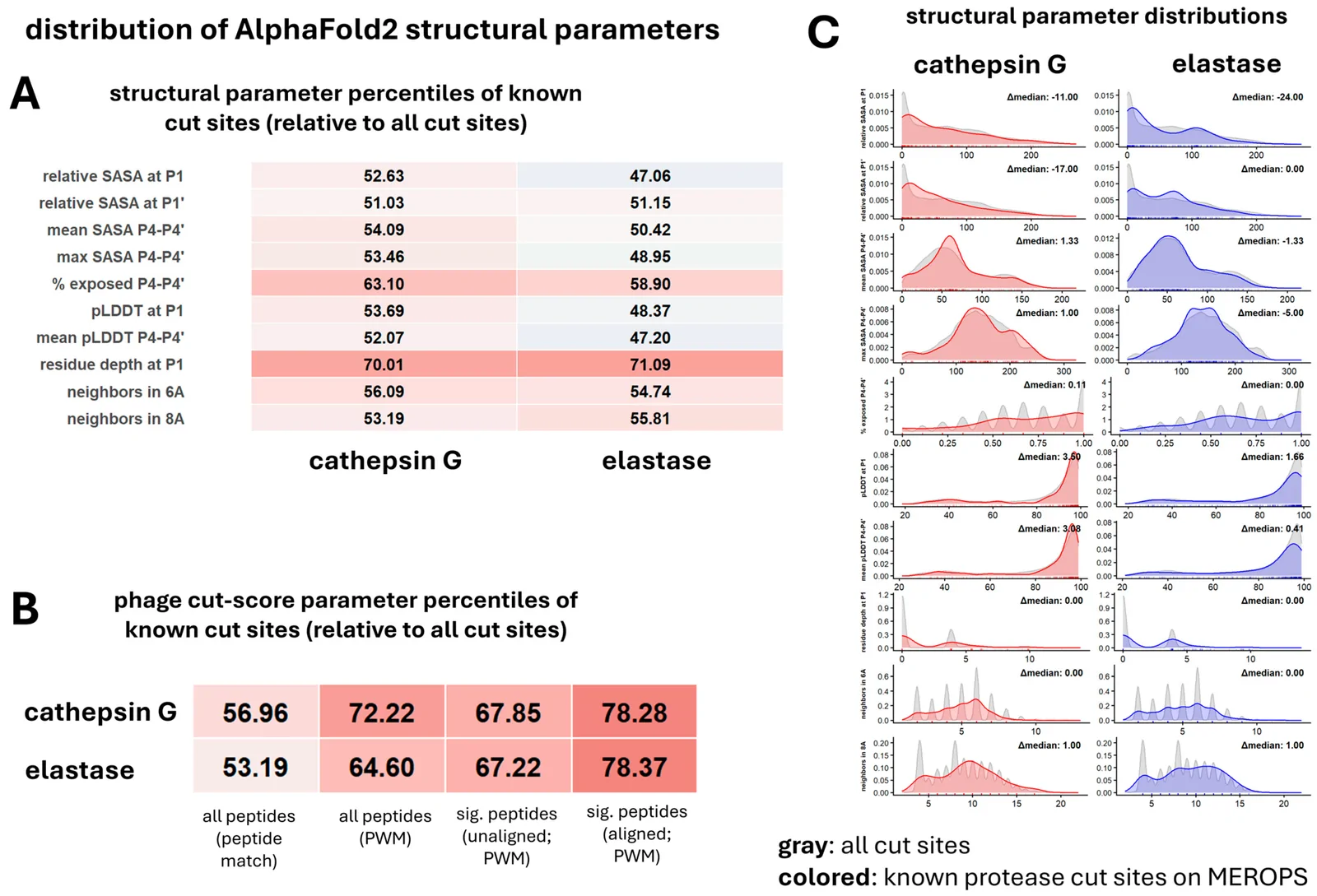
Visualization of structural parameters derived from AlphaFold2 substrate structures. (**A**) Percentiles of structural parameters for known MEROPS cut sites, relative to all cut sites across known MEROPS substrates. Higher percentile corresponds to higher metrics being linked to cleavage. (**B**) Percentile of phage cut scores for known cleavage sites, relative to all cut sites across known MEROPS substrates. (**C**) Density plots of structural parameters at all possible cut sites (gray) or only known MEROPS cut sites (colored).

We next compared sequence-derived signatures with structural features associated with cleavage, including solvent accessibility, residue depth, molecular crowding, and flexibility (pLDDT). Known cleavage sites were ranked by their percentile relative to all possible cut sites, where higher percentiles indicate that cleavage sites were associated with higher values. Among structural parameters, P1 residue depth and solvent exposure across the P4-P4’ region (percentage of positions with SASA > 25) were most strongly associated with cleavage, with known cleavage sites ranking 70.01/71.09 and 63.1/58.90 for cathepsin G/elastase, respectively (Figure 5A). Known cleavage sites also showed clear rightward shifts, compared to background cut sites, for greater solvent accessibility across P4-P4’ (averaged) and P1 or P1’ (Figure 5C). However, these structural features alone provided weaker discrimination than sequence-derived scores, with PWM-based scoring using aligned significant peptides having a percentile of 78.28 and 78.37 for cathepsin G and elastase, respectively (Figure 5B).

Notably, PWM-based scoring outperformed direct matching to individual enriched peptides (peptide match scores) with percentiles of 78.28/78.37 and 56.96/53.19 (Figure 5B) for cathepsin G and elastase, respectively. Scores using aligned significant peptide PWMs outperformed scores using unaligned significant peptide PWMs or PWMs using all peptides, with percentiles of 78.28/78.37, 67.85/67.22, and 72.22/64.60, respectively. Violin plots demonstrated similar results (Figure S14).

### 2.4. Phage-Derived Features Improve Cleavage-Site Classification Compared with Evaluated AlphaFold-Derived Structural Descriptors

To evaluate the contribution of phage-derived scoring relative to AlphaFold-derived structural features evaluated in this study, we trained classification models using XGBoost decision trees to predict cleavage sites, either using structural features alone, phage-derived features alone, or both feature sets combined. To assess baseline performance, we first evaluated models in a balanced dataset of 1:1 known MEROPS cleavage sites to randomly selected cleavage sites. To prevent data leakage between training and test data, cleavage sites from highly similar substrates (CD-HIT 70% threshold for primary sequence similarity) or identical surrounding amino acids (P4 to P4’) were analyzed, with 133 and 212 cut sites filtered out. Training and testing data utilized a substrate-level split, followed by 5-fold cross validation.

**Figure 6:**
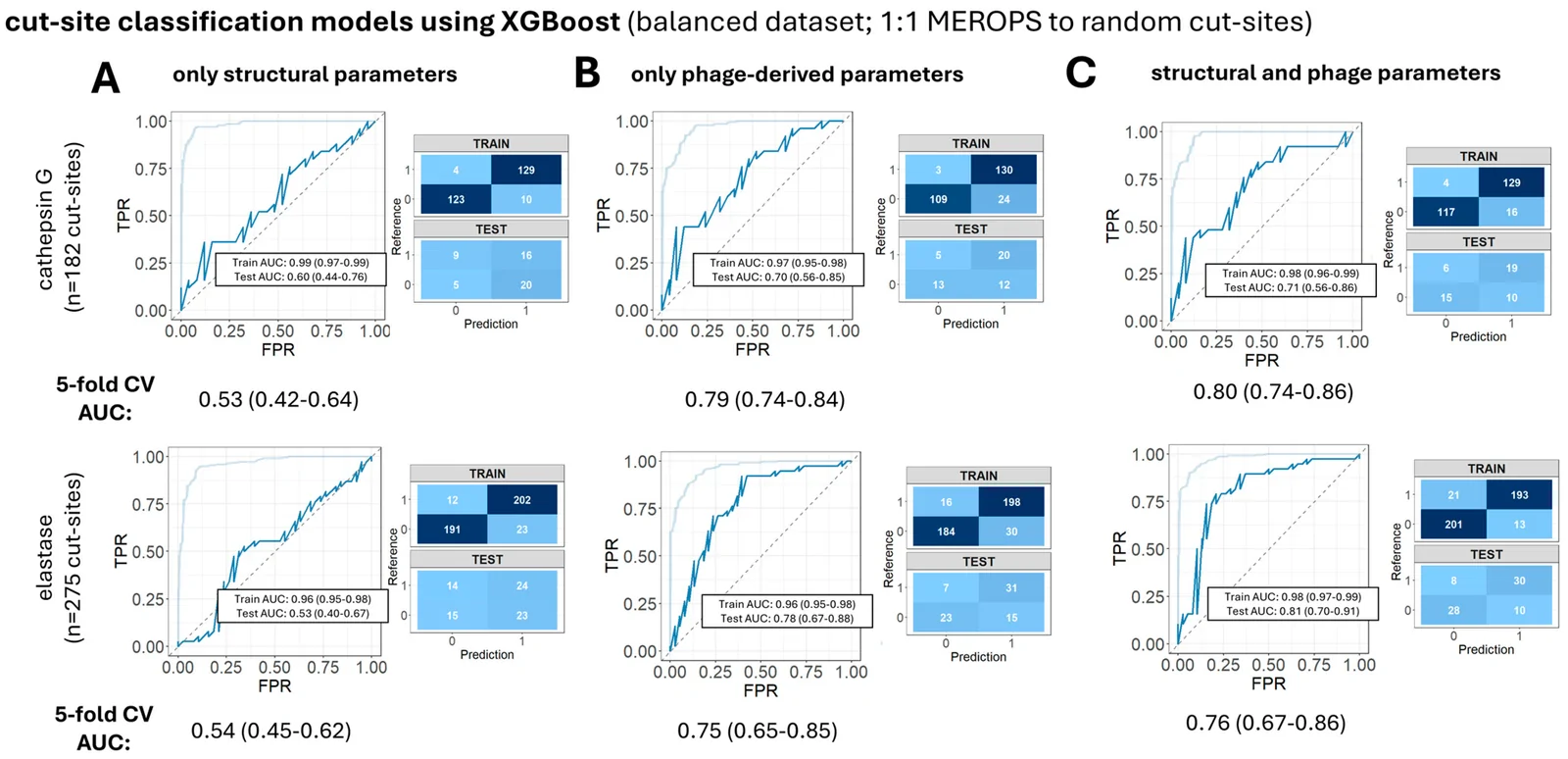
Classification models demonstrate the value of phage-derived scoring for cut site classification, beyond structural parameters. XGBoost machine learning models were trained on known MEROPS cut sites of proteases using an 80-20 train-test substrate-level split. To ensure conservative performance estimates, highly similar cleavage sites were excluded. Models were trained with default parameters (rounds = 100, max depth = 6), except eta = 0.01, subsample = 0.7, and column sample = 0.7 to minimize overfitting. To evaluate comparative performance of structural and phage-derived metrics, models were trained separately on (**A**) structural parameters alone, (**B**) phage-derived parameters alone, and (**C**) both integrated together.

Models based solely on evaluated structural features showed limited predictive power for both cathepsin G (test-set AUC 0.600, 95%CI: 0.440–0.760; AUPRC 0.460) and elastase (AUC 0.533, 95%CI: 0.399–0.666; AUPRC 0.528) (Figure 6A; Table S4). In contrast, phage-derived sequence scores substantially improved performance for cathepsin G (test-set AUC 0.702, 95%CI: 0.566–0.848; AUPRC 0.708) and elastase (0.775, 95%CI: 0.667–0.883; AUPRC 0.731) (Figure 6B; Table S4). Integrating structural features with phage-derived scores produced modest, but not significant, improvements in overall prediction for elastase (AUC 0.806, 95%CI: 0.701–0.911; AUPRC 0.776) and cathepsin G (AUC 0.710, 95%CI: 0.564–0.857; AUPRC 0.728), relative to phage-derived features alone (Figure 6C). Notably, when structural and phage-derived features were combined, phage-derived features were among the top two most important features of the model (Figure S15).

Following the selection of thresholds based on training data, the highest MCC was seen for elastase where phage and structure were integrated (MCC 0.527), compared to 0.365 for cathepsin G (Table S4). Phage feature-only models had the highest sensitivity, rather than phage and structure combined, for both cathepsin G (0.800 vs. 0.760) and elastase (0.816 vs. 0.789), though with the tradeoff of specificity (cathepsin G: 0.520 vs. 0.600; elastase: 0.737 vs. 0.605). Most values stayed stable without significant changes following 5-fold cross validation. Interestingly, while cross-validated metrics mildly decreased for elastase, an increase was seen in cathepsin G test-set AUCs (phage-only: 0.702 to 0.791; phage and structural: 0.710 to 0.803), along with AUPRC and MCC (Table S3). This may be due to differences in substrate composition between the test set and cross-validated folds across the substrate-level splits, such as cross-validated folds having had more informative training substrate data or the fixed test set having comparatively more unconventional substrate cleavage sites.

When evaluating performance across all potential cut sites, emulating a broader real-world screening environment, models based solely on the evaluated structural descriptors again showed limited discrimination (test-set AUC 0.545, 95%CI: 0.426–0.665 for cathepsin G, 0.509, 95%CI: 0.411–0.607 for elastase) (Figures S16 and S17). Phage-derived scores provided moderately strong discrimination (AUC 0.736, 95%CI: 0.621–0.850 for cathepsin G, 0.767, 95%CI: 0.700–0.833 for elastase). A combination of structural and sequence features yielded the best performance for elastase (AUC 0.746, 95%CI: 0.676–0.816) but not cathepsin G (AUC 0.726, 95%CI: 0.620–0.832) (Figures S16 and S17). In parallel to unfitted models, XGBoost results saw low AUPRC values due to the high class imbalance, with phage or phage-structural models having a 3.7- to 5.1-fold or 2- to 2.3-fold higher AUPRC than cathepsin G’s (0.29%) or elastase’s (0.40%) baseline cut site prevalence in the evaluated dataset (Figure S15, Table S5). In phage-structural models, phage-derived scores ranked higher in importance over structural features, especially those from aligned PWMs. All models had a tendency toward specificity over sensitivity, with the highest sensitivity and PPV seen in cathepsin G’s phage-only model (0.520; 0.6%) and elastase’s phage-structural model (0.342; 0.6%). Similar to the balanced dataset, 5-fold cross-validated metrics were stable for elastase, but AUCs and AUPRCs were moderately higher for cathepsin G (Table S5).

## 3. Discussion

Identification of protease cleavage sites in substrates remains a key component in understanding biological systems and disease progression. Altered proteolytic cleavage can alter protein activity, localization, stability, and signaling, such as in cancer, where overactive matrix metalloproteases break down extracellular matrices for tumor spread [23], neurodegenerative conditions, where impaired proteolysis results in protein misfolding or aggregation [24], and dysregulated protease-inhibitor networks contributing to autoimmune or chronic inflammatory responses [25]. Engineered protease inhibition or promotion strategies are emerging as potential pathways to help treat disease [26,27]. While several computational tools have been developed to predict cleavage sites within substrates, many rely heavily on previously annotated substrates or curated databases. This dependency can introduce biases toward well-studied enzymes and limits the ability to characterize proteases under complex physiological conditions, such as in the presence of inhibitors, modulators, or newly discovered protease variants.

Here, we present a complementary approach that integrates de novo substrate phage display experimental data for cleavage site prediction, using scoring methods to rank and prioritize P1-P1’ cleavage sites across proteins. Our results demonstrate several key findings. First, phage-derived sequence scores alone exhibited predictive power, even without additional fitting or model optimization. Across both balanced datasets (known cleavage sites matched with random cut site controls) and global screening contexts (evaluation across all potential cut sites in substrates), PWM-based scoring achieved moderately strong discrimination between cleaved versus background unannotated sites for cathepsin G and elastase. This suggests that experimentally derived sequence scores captured determinants of protease specificity and can be applied directly for cleavage site prediction in the absence of curated substrate databases. These findings were corroborated with phage-derived scoring distributions showing clear rightward shifts for known cleavage sites compared with background sites across substrates, indicating that sequence scores were associated with cleavage-site likelihood. Furthermore, experimentally derived PWMs consistently outperformed 10,000 randomly generated PWMs when evaluated against known cleavage sites, supporting the biological relevance of observed sequence signatures.

Second, combining complementary specificity representations can improve cleavage-site prediction, but the optimal representation appears protease-dependent. For cathepsin G, combining PWM scores derived from both significant peptides and all peptides enhanced discrimination of cleavage sites more than significant peptides alone, in both balanced and global cut site situations. However, this improvement was not observed for elastase, indicating that the benefit of integrating broad and specific sequence representations may depend on protease-specific substrate preferences. As such, while substrate phage display provides both generalized sequence motifs and individual peptide-level information, the relative utility of these representations may vary across proteases. Third, classification models using phage-derived sequence scores consistently outperformed models using the evaluated structural parameters. Although the primary goal of this study was to establish a database-independent cleavage screening strategy to generate protease-specific features, rather than a trained predictive model, XGBoost classifiers provided a useful framework to evaluate the relative contribution of different features. In these models, test-set classification performance was highest in models using phage-derived sequence scores than in those using the selected structural features alone, for both proteases. This indicated that sequence determinants captured by the phage screen provided stronger predictive signals than structural accessibility metrics. Nonetheless, compared to sequence scores alone, combining sequence and structural features modestly improved prediction performance for both cathepsin G and elastase in balanced contexts, though not in global contexts. As such, structural accessibility may provide complementary information that may further refine cleavage site prediction. Future integrations with deep-learning models (e.g., AlphaFold or ESMFold) and emerging protein–protein interaction algorithms could further refine this framework, providing scalable, database-independent structural metrics and features for cleavage site prediction.

Importantly, the substrate preferences identified in this study were consistent with previously reported biochemical and structural data for neutrophil serine proteases. Cathepsin G displayed enrichment for large aromatic (F/Y) and small hydrophobic residues (L/M) in the P1 position, consistent with its deep hydrophobic S1 pocket [28]. Elastase favored smaller aliphatic residues (V/I/L/A) at P1, consistent with a shallow hydrophobic S1 pocket [29]. Extended subsite preferences beyond P1 further support agreement, such as serine at P1’ for cathepsin G and elastase, and aspartic acid or glutamic acid at P2’ for cathepsin G [30,31]. Other observed preferences were also consistent with the previous literature, including V/L at P1’ for cathepsin G and T at P1’ for elastase [28,29].

We compared our score’s performance to ProCleave, a cleavage site prediction tool that integrates sequence and structural information using a conditional random field framework (CRF) [32]. ProCleave was trained on MEROPS cut sites and evaluated in a balanced setting, with negative sites randomly selected to match the number of MEROPS sites. For elastase, our model demonstrated moderately strong zero-shot generalizability; despite not being trained or fine-tuned on any MEROPS data beforehand, PWM scores from aligned significantly cleaved peptides achieved an AUC of 0.803, or an AUC of 0.787 when normalized within the substrate to 0–1, which closely matched ProCleave’s 0.818. However, for cathepsin G, ProCleave maintained a higher AUC of 0.891. Our untrained scores initially reached an AUC of 0.783, which improved to 0.803 in our trained model under 5-fold cross validation. Apart from ProCleave, other cleavage prediction tools were examined, but limited data exist on their validation for cathepsin G and elastase. As such, no performance comparisons could be made.

Overall, we developed PhageScout, an experimental platform for de novo protease substrate site specificity data, demonstrating the capacity to capture protease-specific features and predict cleavage sites in substrates without prior substrate knowledge or existing database entries. In particular, this framework can capture cleavage site trends across multiple resolutions, from broad motifs to fine-grained peptide details. We have shown that this dual-level scoring may enhance cleavage site prediction performance, though in a setting-specific way. We theorize that while PWMs capture broad probabilistic preferences of a protease, significant peptides represent dominant cleavage cases, and specific peptide matches allow models to account for rare sequence interactions lost in matrix-based averaging. Furthermore, we demonstrated that phage-derived scores outperform the selected traditional structural parameters in discriminating cleavage sites, but as observed in our trained models, integrating both feature types may enhance performance in balanced comparison settings.

Several limitations should be acknowledged. The peptide library sampled approximately 29.7% of the theoretical 20^5^ sequence space, leaving potential substrate sequences unrepresented. Displayed phage peptides are also short and linear, lacking the structural context of native protein substrates. In addition, cut sites within 5-mers remain uncertain and require inference using sliding window scoring or unsupervised alignment. This limits the positional information available for scoring, requiring an averaged 5-mer PWM across possible cleavage positions, which may dilute specific positional preferences and reduce the strength of the resulting PWM signal. This is inherent to substrate phage display, where the precise cleavage position within the displayed peptide is not directly known. The limitation was partially mitigated through unsupervised alignment to infer a consensus cleavage position and P1 site, which improved performance, but this step remains an inference.

Furthermore, the present study evaluated only two neutrophil serine proteases, and benchmarking was limited to substrates annotated in MEROPS. Benchmarking methods were consistent with common validation strategies, and cathepsin G and elastase have distinct but partially overlapping substrate specificities, providing a useful test case for whether PhageScout can distinguish protease-specific cleavage site preference. However, broader evaluation across additional proteases and physiological substrates will be important to further assess generalizability. In particular, the performance across protease classes with different substrate recognition mechanisms, including cysteine, aspartic, and metalloproteases, remains unknown. Proteases whose substrate recognition depends more strongly on secondary structure, tertiary context, or longer-range sequence interactions, such as proteolytic complexes or multidomain regulatory proteases, may be less readily evaluable by phage display, and the utility of PhageScout for these proteases remains to be established. The structural features we evaluated also represent a limited set of descriptors and a simplified method that may not capture cleavage site preferences as strongly compared to other established structure-based cleavage site prediction methods.

Current findings also only provide retrospective validation against previously annotated cleavage sites, rather than prospective biochemical validation of novel predicted sites. Prospective biochemical validation of a panel of novel high-scoring sites will be necessary to determine whether PhageScout can reliably identify previously unannotated cleavage sites. Additionally, unannotated positions in MEROPS-associated substrates were used as background sites. Yet, these positions cannot be assumed to be definitively non-cleaved; some may represent experimentally uncharacterized cleavage events (false-negatives). Accordingly, our classification results may reflect discrimination between annotated cleavage sites and background/unannotated sites, rather than definitive positive-versus-negative cleavage.

Despite these constraints, our approach provides a database-independent framework for generating protease-specific features to help predict protease cleavage sites without prior substrate knowledge. This may be particularly useful for studying poorly characterized proteases, variants, or proteases operating in complex physiological environments. Future work will expand this platform to additional protease classes, refine scoring methods to improve detection of cleavage determinants, and support prospective biochemical validation for novel cleavage site discovery.

## 4. Materials and Methods

### 4.1. Phage Library Development and Screening

A substrate M13 bacteriophage display library was generated using the fUSE67 phage vector, featuring a randomized 5-amino acid (5-mer) peptide insert flanked by a FLAG tag on the pIII protein (Figure 1). The library was constructed via NNK saturation mutagenesis using 10 ng of oligonucleotide template (L1) and 1 µM primers (S1/AS1), as previously described [18]. The NNK degenerate codon series involved 15 nucleotides for 5 amino acids, with N having equal proportions (25%) for A/C/G/T, and K having 50% proportions for G/T. PCR products (30 cycles: 95 °C, 60 °C, 72 °C for 30 s each) were digested with AscI and NotI, gel-purified, then ligated into 1 µg fUSE67 at a 3:1 molar ratio (insert:vector). The product was electroporated into MegaX DH10B *E. coli* and titrated on tetracycline LB agar. The resulting 1 × 10^7^ independent clones provided >3-fold coverage of the expected 20^5^ unique 5-mer sequences [33].

For each screen, phage clones were grown overnight in LB (30 µg/mL tetracycline), immobilized on 50 µL anti-FLAG agarose beads (Sigma-Aldrich, Burlington, MA, USA) and mixed at room temperature for 2 h, pelleted by centrifugation (1 min 2000× *g*) and washed five times with TBS-T to remove unbound phage. Immobilized phages were incubated with commercially obtained 10 nM cathepsin G (6 h) (Sigma) or 5 nM neutrophil elastase (Enzo Life Sciences, Farmingdale, NY, USA) (1.5 h). Incubation times and concentrations were calibrated to prevent non-specific backbone cleavage, as determined by ELISA [18,19]. To control for baseline phage composition, the unselected phage library was obtained through elution with 5 mg/mL of 3× FLAG peptide (2 h) [33]. All reactions were performed in biological triplicates.

#### ELISA Screening for Phage

To assess protease-dependent cleavage of immobilized phages, an ELISA-based assay was performed to quantify uncleaved phages retained on anti-FLAG agarose beads. Immobilized phages were exposed to either increasing concentrations, increasing durations, or both for cathepsin G and neutrophil elastase. Following incubation, residual uncleaved phages retained on the beads were quantified by ELISA, with absorbance measured at 450 nm. Experiments established optimal incubation durations for each enzyme. Thresholds were selected as the latest timepoint before a >30% reduction in immobilized phages was observed, thereby minimizing potential non-specific cleavage while maintaining sufficient enzymatic activity.

### 4.2. DNA Preparation and Next-Generation Sequencing

Supernatants containing cleaved phages were collected, and single-stranded DNA (ssDNA) was extracted using proteinase K followed by phenol/chloroform/isoamyl alcohol (Invitrogen, Carlsbad, CA, USA) and isopropanol precipitation. Purified ssDNA underwent two rounds of PCR (95 °C, 60 °C, 72 °C for 30 s each) with cycle numbers minimized to prevent over-amplification. PCR products were gel-extracted, quantified by Picogreen dsDNA assay (Invitrogen), and sequenced (paired-end 100 bp) on an Illumina NextSeq 2000 platform [18,33,34,35]. The PicoGreen dsDNA assay involved diluting samples in TE buffer and mixing with an equal volume of PicoGreen working solution and being incubated for 2–5 min at room temperature protected from light. Fluorescence was measured at approximately 480 nm excitation and 520 nm emission, with DNA concentrations determined relative to a dsDNA standard curve.

Paired-end reads were assembled using PEAR with a quality score threshold of 20, where reads with consecutive bases below PHRED 20 were removed [36]. Assembled reads were oriented to one of four 8 bp seed sequences and trimmed to isolate the 5-mer mutagenesis region. Following translation and merging, peptides containing stop codons or displaying low abundance (counts < 10 across all conditions) were excluded. To account for baseline phage and NNK residue composition, we utilized DESeq2 (v1.50.2) [37] to calculate log2-fold change (L2FC) of peptide enrichment relative to the unselected control library (FLAG peptide elution). DESeq2 involved scaling samples by size factors (median-of-ratios) and fitting a negative-binomial model per peptide to estimate changes and *p*-values for each protease, versus FLAG control. Downstream analysis was conducted with Python 3.11 [38] and R v4.3.1, as described below [39].

### 4.3. Scoring Potential Cleavage-Sites Using Phage-Derived Protease Preference Data

To predict cleavage sites within substrates, we adopted a site-level approach that screens and scores every possible P1 cut site in a protein (Figure 2). Similar motif-based strategies have previously been used to predict protease cleavage sites from experimentally derived substrate preferences, including approaches based on position weight matrices (PWMs) and related specificity models [40]. Our method differs by deriving these substrate preference profiles directly from unbiased substrate phage display selections, enabling protease-specific scoring from experimentally observed cleavage site preferences.

From our phage library protease screen, cleaved phages represent specific peptide sequences that each protease cut and subsequently recorded from next generation sequencing, with their relative enrichment corresponding to the extent of substrate preference. By analyzing these sequences, we created protease-specific cleavage site preference profiles as a fingerprint of amino acid patterns each protease prefers. Because a single protease may recognize both broad patterns and highly specific sequences, we utilized two distinct scoring methods: position weighted matrices (PWMs) and peptide match profiles.

The PWM method involved a 5 × 20 matrix of amino acid probabilities, where each amino acid of each 5-mer cleaved peptide contributed to this probability. As such, the probability of each amino acid *a* at a position *j* was denoted by $P_{aj}=\frac{count\;of\;a\;at\;j}{number\;of\;peptides}$. To ensure our results reflect protease preference, we weighed these probabilities by their log2-fold change relative to the unselected library, such that more abundant cleaved peptides contributed more to the count. The final PWM, in log-2 space, was defined as ${PWM}_{aj}={log}_{2}\left(\frac{P_{aj}}{FLAG\;control\;frequency\;of\;a\;at\;j}\right)$. For each cut site, a score was calculated as the sum of probabilities from matching amino acids of the substrate (${PWM}_{score}=\sum_{j=1\;to\;5}{PWM}_{aj})$ or (${PWM}_{score}=\sum_{j=1\;to\;9}{PWM}_{aj})$ if aligned. To capture a more refined and specific signature, we also created a stricter version of this matrix that only included peptides that passed a conservative statistical threshold (Bonferroni-corrected *p*-value < 10^−3^ and log2-fold change > 1 from DESeq2) for significance (significantly cleaved peptides).

One technical challenge is that while we know the 5-mer was cut, we do not know exactly where the cut occurred. To account for this uncertainty, a sliding window approach was applied, where we scored all four possible ways a 5-mer could overlap with the cut site, and subsequently averaged the summed scores over four for a single final score. This score was then normalized on a scale of 0 to 1 per substrate, allowing enhanced comparisons of cut site scores across substrates ($a=\frac{x-\min x}{\max x-\min x}$), where x is the score and a is the 0–1 normalized score per substrate.

As an alternative solution to this uncertainty, unsupervised alignment was also applied using DECIPHER (v11.38) [41] to align peptides to a dominant amino acid that serves as the primary cleavage site determinant, which maps all 5-mers in a 9-mer space without any prior substrate or alignment knowledge. DECIPHER uses sequence similarity of the 5-mer primary sequences, shifting each 5-mer to the left or right by up to 4 positions, building a P5-P4’ consensus alignment motif of all peptides with the goal of maximizing overlap. The presumed P1 position was assigned to the DECIPHER-aligned position exhibiting the strongest predominant amino acid signal, based on the assumption that P1 represents the major determinant of substrate site specificity. This approach has been demonstrated in another study [17] and allows a PWM score to be generated without sliding windows.

To complement the broad patterns found by PWM scores, we also used a peptide match profile. Rather than calculating general probabilities, this method more directly uses the exact enrichment scores (log2-fold change) of cleaved peptides from our screen. Again, we used a sliding window to average the scores of all possible 5-mers covering a cut site, as denoted in Figure 2. This approach captured specific peptide-level preferences by the protease. For 5-mers, the peptide match score is therefore defined as the mean of DESeq2 log 2-fold change levels across the 4 possible sliding windows where the 5-mer could be on the substrate.

PWMs and scoring distributions were visualized using ggplot (v4.03) and ggseqlogo (v0.2.2) [42,43,44,45]. Code for processing and analysis, along with normalization or optimization parameters, is available on GitHub (https://github.com/YuEnoch/PhageScout, accessed on 9 August 2026) and Zenodo (https://zenodo.org/records/21387981, accessed on 9 August 2026).

### 4.4. Evaluation and Validation Classification Performance Using Phage-Based Cut Scores

To benchmark the performance of these scores, we evaluated predictions against all known cleavage sites from the MEROPS peptidase database [30], a gold-standard collection of protease substrate cut sites used by multiple studies for protease cleavage prediction [5,13,14], that were also present with an AlphaFold PDB structure available [46]. We identified 164 and 188 unique human substrates for cathepsin G and neutrophil elastase respectively, corresponding to 265 and 347 known cleavage sites (Table S1). These cut sites were visualized using ggseqlogo (Figure S1). Eligible cut sites included any cut site with a P4-P4’ sequence of only natural amino acids. Sequences with synthetic amino acids were excluded.

To evaluate classification performance against a realistic baseline of unannotated background sites, we pooled all substrates across the two enzymes (270 substrates; 120,287 possible cut sites) as a comprehensive background (‘full dataset’). Analyses were also repeated in a balanced context (‘balanced dataset’), where cut sites were instead matched to random background/non-annotated sites, to directly evaluate discriminative power. This was tested through density plots comparing scoring distributions of cleaved versus background/non-annotated sites, and receiver operating characteristic curves (ROCs) or precision-recall curves (PRCs) for classification, using pROC [47]. The 95%CIs for area under ROCs (AUC) or PRCs (AUPRC) were estimated using DeLong’s method or bootstrapping with 1000 rounds. Confusion matrices were generated with an ideal threshold determined using Youden’s method, followed by the calculation of sensitivity, specificity, PPV, NPV, and MCCs with 95%CIs using a binomial distribution.

To further evaluate the biological signal in PWMs, we generated 10,000 randomized 5-mer and 9-mer PWMs to score all known MEROPS cut sites. If phage-derived PWMs carry biologic information for predicting protease cut sites, we expected the experimentally derived PWMs to yield higher scores for validated cut sites, compared to other randomized PWMs.

### 4.5. Integration of Protein Structural Parameters for Cleavage Classification

While sequence motifs provide a blueprint for protease specificity, they do not account for structural parameters of a folded substrate, such as an accessible non-buried target site or molecular crowding around the site. We aimed to determine which 3D structural features are relevant in defining a cleavable site and, crucially, if our phage-derived sequence scores have utility in enhancing these structure-based predictions.

To capture this physical context, we integrated 3D structures of all 270 substrates derived from AlphaFold2, sourced as PDB files [46]. We focused on metrics of exposure and flexibility, including six measures of solvent accessibility: relative solvent accessible surface area (SASA) at P1 and P1’ positions, maximum and mean relative SASA within the P4-P4’ window, and percentage of exposed sites within that same window (relative SASA > 25) [48,49,50]. We also evaluated model confidence (pLDDT) as an indirect measure of disorder, at P1 specifically and as a mean across P4-P4’, the number of residues within a 6 or 8 Ångström radius as a measure of molecular crowding, and the type of secondary structure at either P1 or P1’ (as helix, loop/coil, or sheet). We visualized trends using density plots and percentile parameters to compare structural parameters between cleaved and background non-annotated sites from MEROPS.

We consolidated these features with our phage-derived scores using an XGBoost machine learning classifier. We designed this framework with two specific objectives: first, to identify which structural or biochemical features are most relevant for cleavage, and second, to test if adding our phage-derived scores adds explanatory power beyond structural data alone. To ensure realistic performance estimates, we trained models in both a balanced dataset and full dataset setting. To prevent data leakage and the model memorizing known targets, we filtered out substrates with highly similar known cleavage sites (exact same P4-P4’) or substrates highly similar to each other (70% similarity threshold by CD-HIT), mirroring existing approaches [14]. A substrate-level split was also used to ensure limited overlap of substrate-specific sequence and structural features between training and test sets.

Models were trained using an 80-20 train-test substrate-level split, for a binary cleavage prediction with an AUC evaluation measure. Default parameters were used (max-depth of 6, 100 rounds), except with a lower learning rate (eta = 0.01) and only showing the model 70% of data at a time (subsampling = 70%, column-subsampling = 70%) to prevent overfitting. We evaluated results using ROC, PRC plots and confusion matrices, with Youden’s method calculated on the training dataset to identify the optimal threshold. The 95%CIs for AUC were estimated using the DeLong method. Subsequently, the XGBoost models were repeated for 5-fold cross validation, using an 80-20 train-test substrate-level split with the same parameters as above. Metrics including sensitivity, specificity, MCC, NPV, PPV, and AUPRC were subsequently calculated with their 95%CI based off their variability across the five folds.

## 5. Conclusions

PhageScout provides a database-independent framework for generating protease-specific cleavage features from de novo substrate phage display and prioritizing potential cleavage sites without relying on prior substrate annotations. Phage-derived sequence scores demonstrated moderately strong discrimination of known cleavage sites from background sites without prior training or prior protease-specific adjustment, while integration with structural features provided modest, context-dependent improvements in prediction performance. Further validation across diverse proteases and prospective biochemical testing of novel predictions will be important to establish the generalizability and utility of PhageScout for cleavage-site discovery.

## Data Availability

This study did not generate new unique reagents. Unprocessed NGS sequencing data have been deposited in the Sequencing Read Archive (SRA), with accession number SRX25506884 (https://www.ncbi.nlm.nih.gov/sra/?term=SRX25506884, accessed on 12 October 2025). All code scripts and processed data files to reproduce figures in the paper have been uploaded to a GitHub repository (https://github.com/YuEnoch/PhageScout, accessed on 9 August 2026) and Zenodo dataset source (https://zenodo.org/records/21387981, accessed on 9 August 2026). Any additional information required to reanalyze the data reported in this work paper is available from the lead contact upon request. Requests for further information and resources should be directed to Colin A. Kretz (colin.kretz@taari.ca).

## Supplementary Materials

The following supporting information can be downloaded at: https://www.mdpi.com/article/10.3390/ijms27177593/s1.

## Abbreviations

The following abbreviations are used in this manuscript:

AUC	Area Under the Receiver Operating Characteristic Curve
AUPRC	Area Under the Precision-Recall Curve
MCC	Matthews Correlation Coefficient
PWM	Position Weight Matrix
MEROPS	MEROPS Peptidase Database
PLM	Protein Language Model
SASA	Solvent Accessible Surface Area
pLDDT	Predicted Local Distance Difference Test
ROC	Receiver Operating Characteristic Curve
ELISA	Enzyme-Linked Immunosorbent Assay
PCR	Polymerase Chain Reaction
NGS	Next-Generation Sequencing
LB	Luria–Bertani broth
TBS-T	Tris-Buffered Saline with Tween 20
FLAG	FLAG Epitope Tag
L2FC	Log_2_ Fold Change
XGBoost	Extreme Gradient Boosting (decision tree library)
NNK	Saturation Mutagenesis Sequence (32-codon library)
PEAR	Paired-End reAd mergeR Software
DESeq2	Differential Expression Analysis Using Sequencing Data (version 2)
PPV	Positive Predictive Value
NPV	Negative Predictive Value
95%CI	95% Confidence Interval
PDB	Protein Data Bank file for 3D Protein Structures
PHRED	DNA Quality Score
ssDNA	Single-Stranded DNA
dsDNA	Double-Stranded DNA
CD-HIT	Sequence Clustering Program to Identify Highly Similar Proteins
ETA	Estimated Time of Arrival (XGBoost parameter)
