# Supplementary material for: PhageScout: Protease Cleavage Site Prediction Using an Experimental Substrate Phage Display Motif-Based Approach
Source: Int J Mol Sci. 2026 Aug 25;27(17):7593. doi: 10.3390/ijms27177593 (PMC13565973; doi:10.3390/ijms27177593)
Supplement: Supplementary file 1 [file ijms-27-07593-s001.zip › ijms-4475996-supplementary.pdf]

Table S1: Distribution of identified substrates and cut sites for cathepsin G and elastase on the MEROPS peptidase database.

	Identified substrates	Identified substrate cut sites	Physiological vs Non-Physiological
cathepsin G	164	265	47 / 218
elastase	188	347	125 / 222
Total	270	581	168 / 413

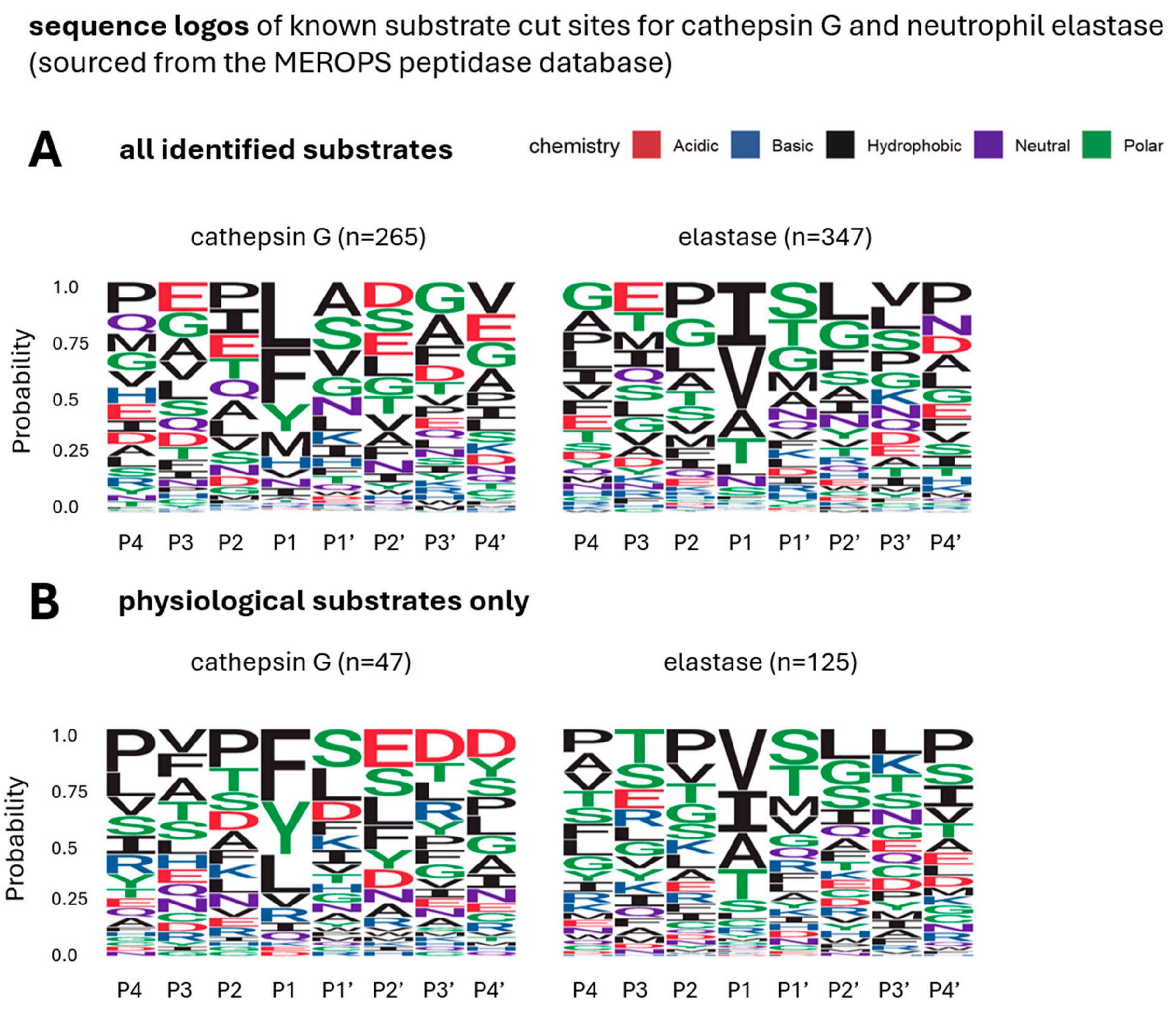

Figure S1: Characterization of known substrates for cathepsin G and neutrophil elastase identified from the MEROPS peptidase database. **A)** sequence logos of specificities for all substrates. **B)** sequence logos of specificities for only physiological substrates.

Table S2: Summary of peptide counts across protease screens from substrate phage display.

	**All peptides screened**	**Significance threshold (log2-fold change >1, p-adj <10^{-3})**
cathepsin G	652,509	9,577
elastase	652,509	8,364

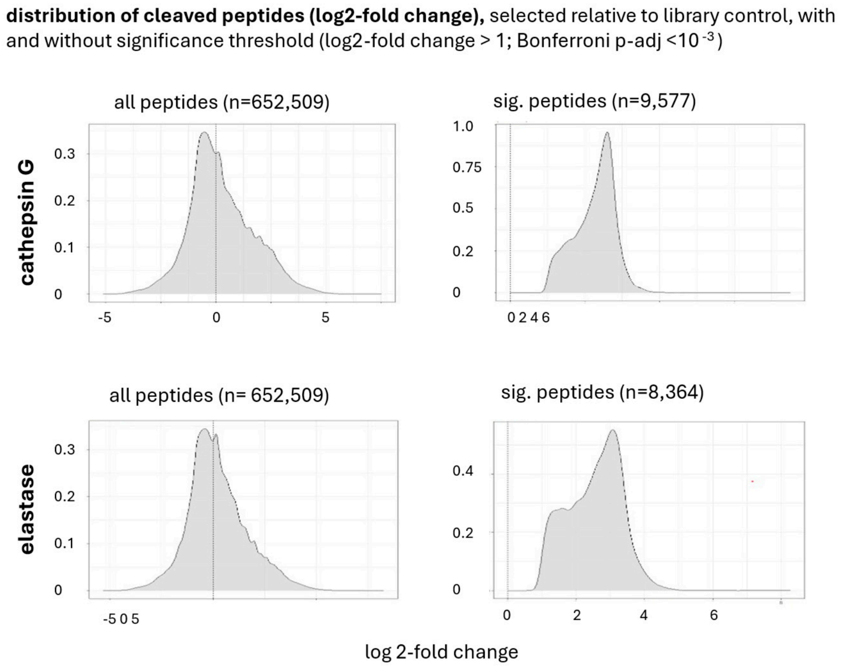

Figure S2: Distributions of peptide enrichment, relative to the unselected phage library, across all peptides or significantly cleaved peptides for each protease.

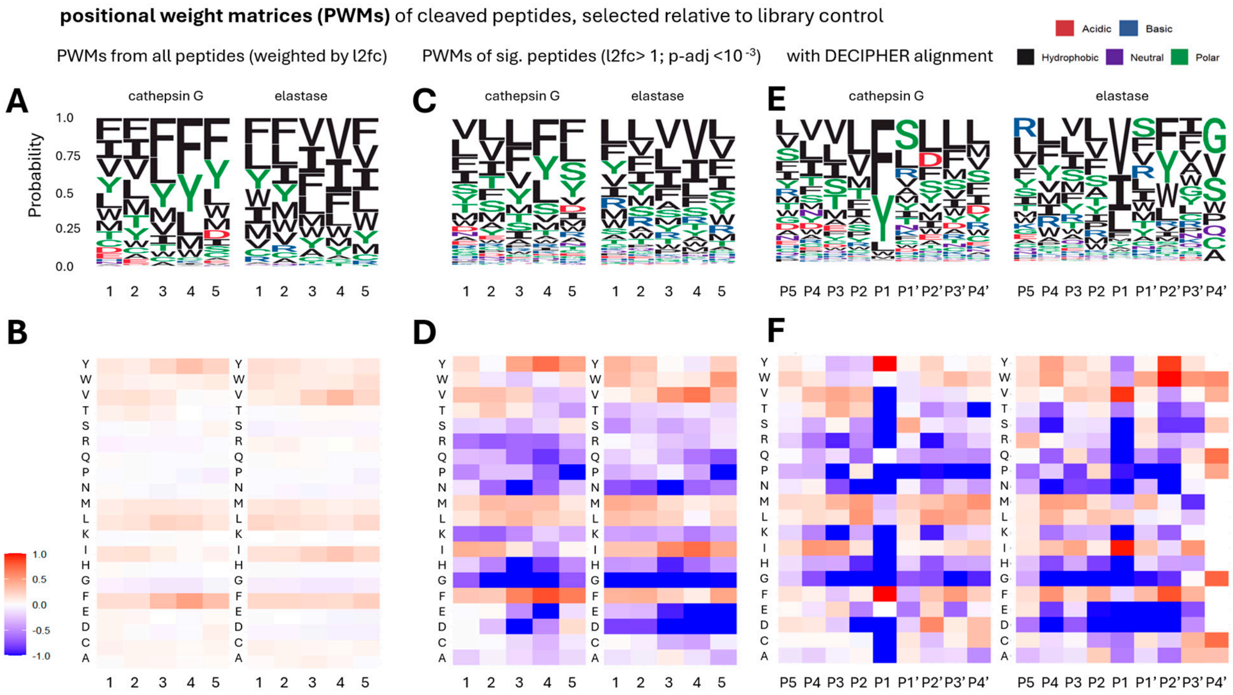

Figure S3: Positional weight matrices (PWMs) of protease cleavage specificity. **A-B)** PWMs of all peptides, each weighed by its log2-fold change to control, as a heatmap or sequence logo. **C-D)** PWMs of significantly cleaved peptides. **E-F)** PWMs of significantly cleaved peptides following unsupervised alignment into 9-mer space by DECIPHER.

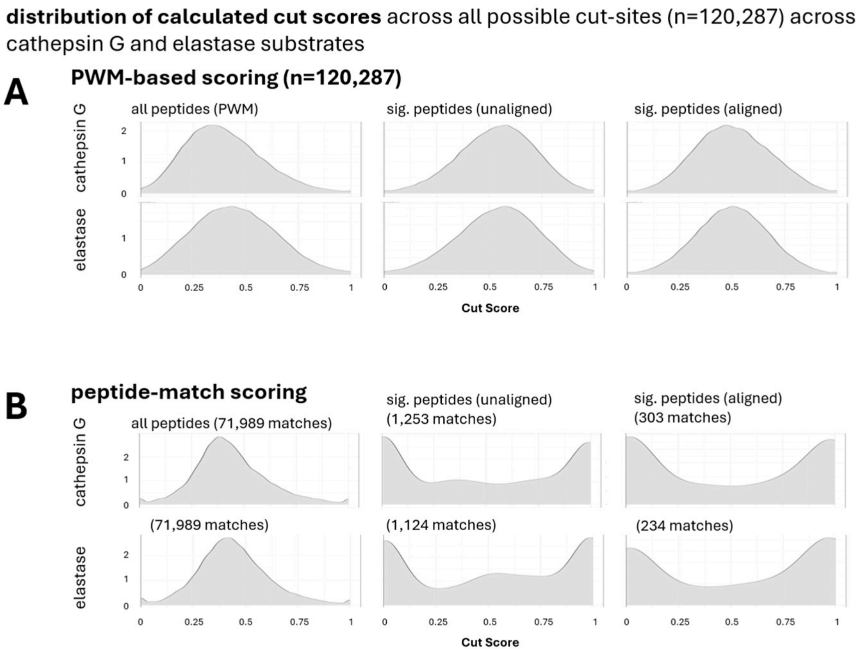

Figure S4: Distributions of phage-derived cut scores (normalized 0 to 1 per substrate) across all possible cut sites in tested substrates, using **A)** PWM-based scoring or **B)** peptide-match based scoring, for the three proteases.

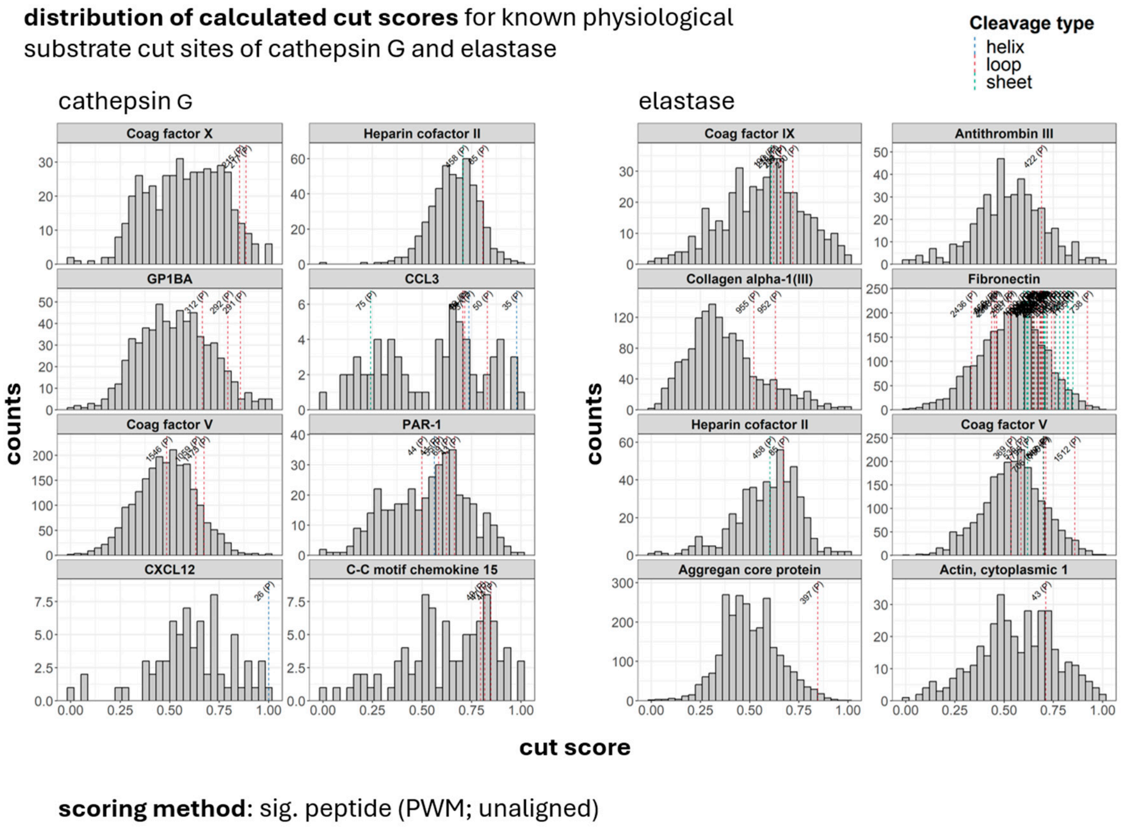

Figure S5: Distribution of phage-derived cut scores for known physiological substrates of cathepsin G and elastase, with a background of all possible cut sites (grey) compared to known cut-sites (colored).

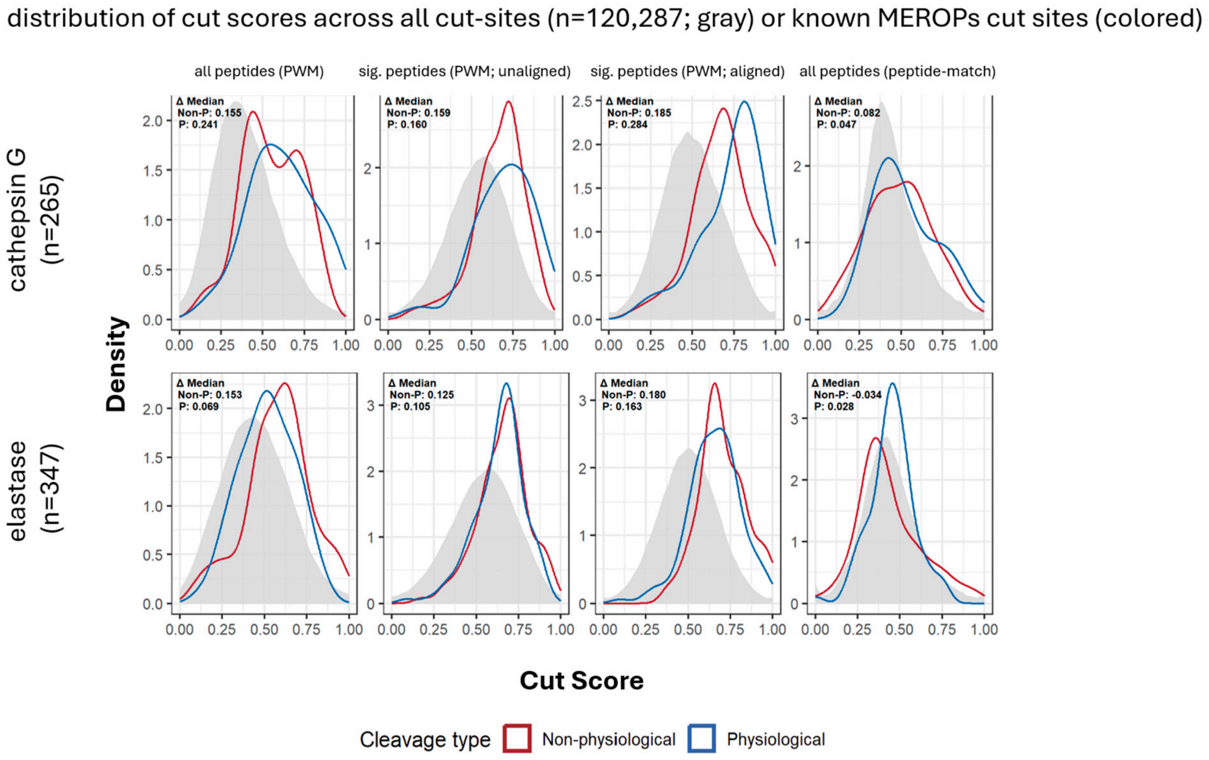

Figure S6: Density distribution of phage-derived cut scores, with a background of all possible cut sites (grey) compared to known cut-sites (colored). Cut sites were subgrouped by non-physiological or physiological cut sites.

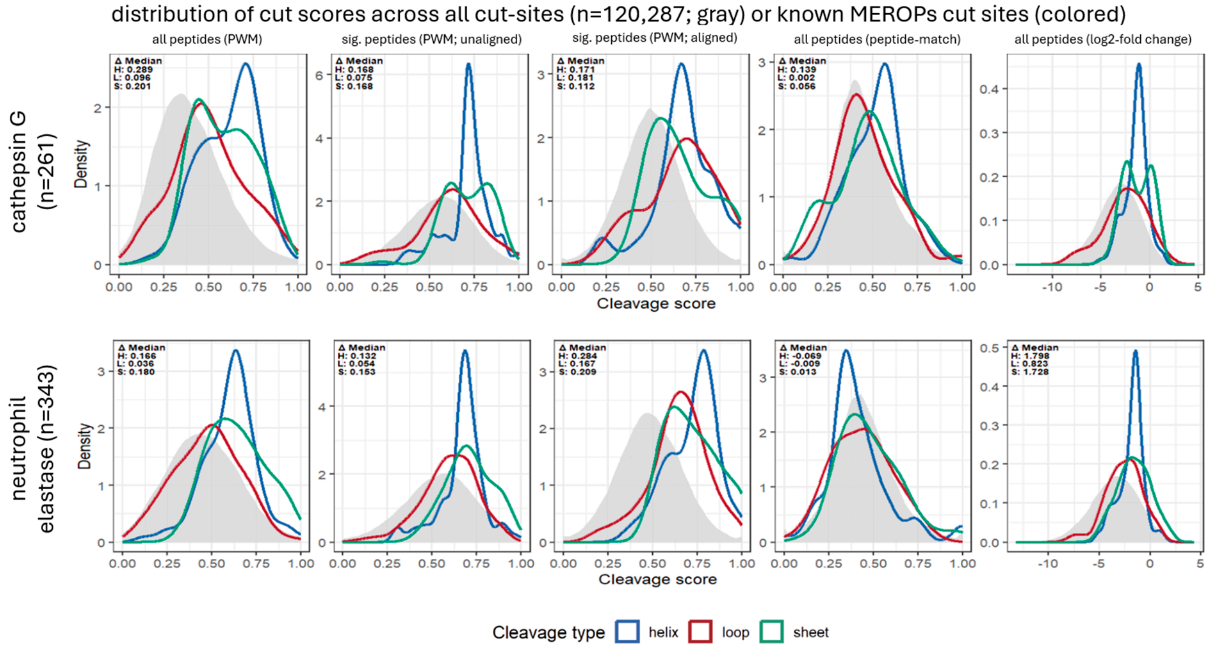

Figure S7: Density distribution of phage-derived cut scores, with a background of all possible cut sites (grey) compared to known cut-sites (colored). Cut sites were subgrouped by cut-site secondary structure.

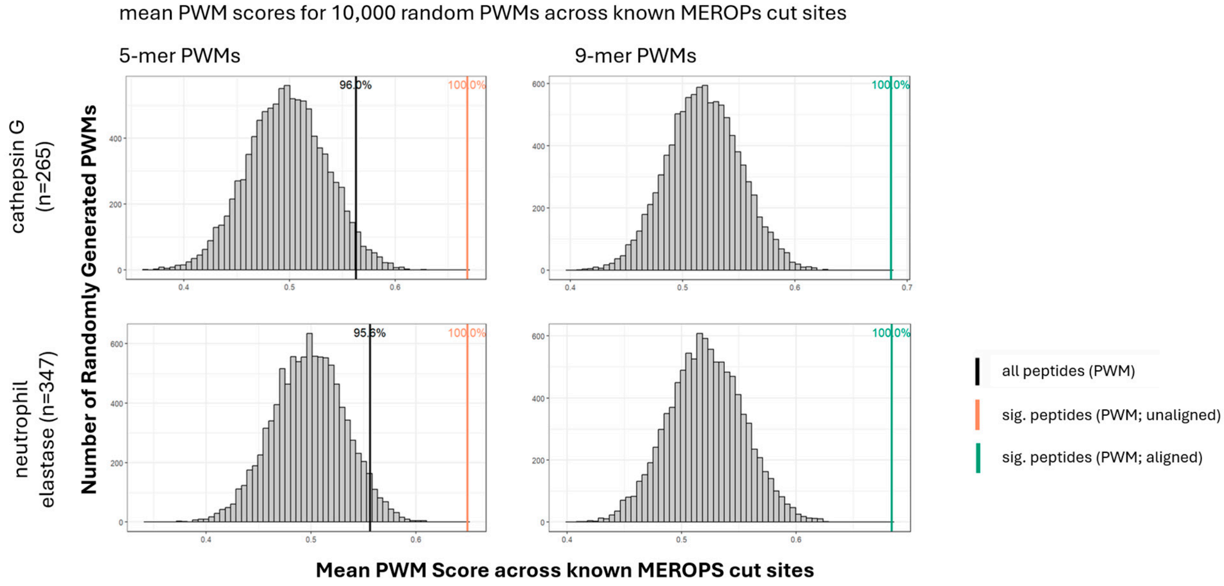

Figure S8: Validation of phage-derived PWMs compared to 10,000 randomly generated PWMs. PWM scores across all MEROPS cut sites were calculated across 10,000 randomly generated PWMs (gray), then to phage-derived PWMs (colored) with percentiles labeled.

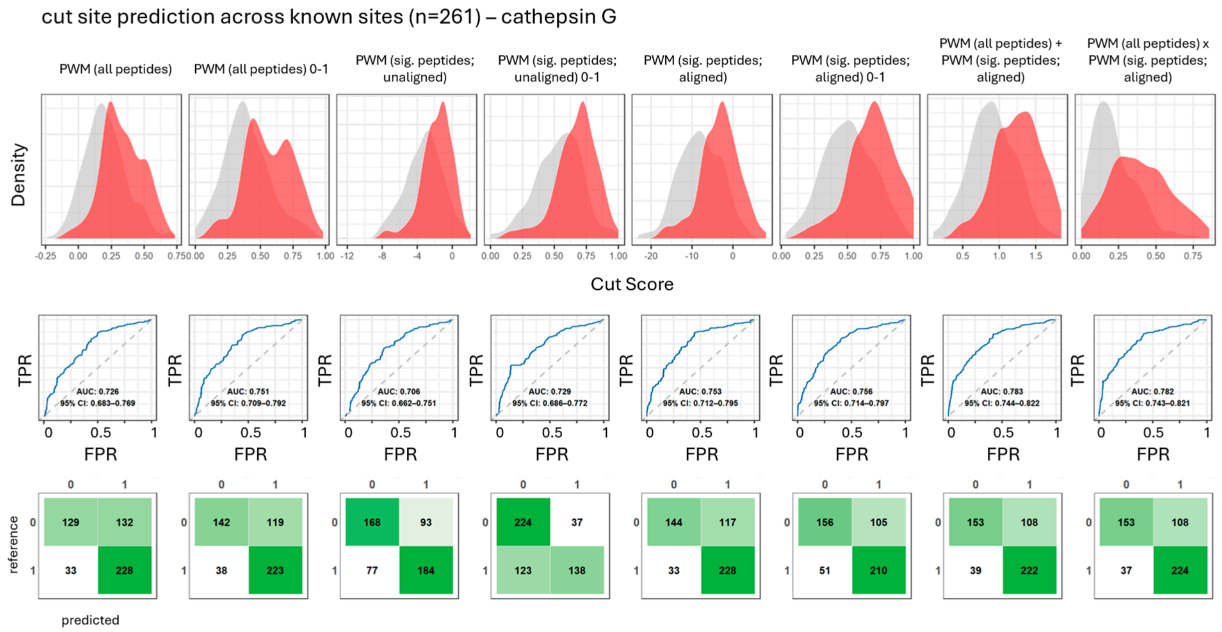

Figure S9: Classification performance of phage-derived cut scores for cathepsin G. Density plots represent scores with a background of all cut sites (grey) or known cut sites (red). ROC curves represent discriminative ability between cleaved and background/unannotated sites, with an ideal threshold determined by Youden's method and used for the confusion matrix. Performance was evaluated in a balanced dataset with a 1:1 ratio of MEROPs to random cut-sites.

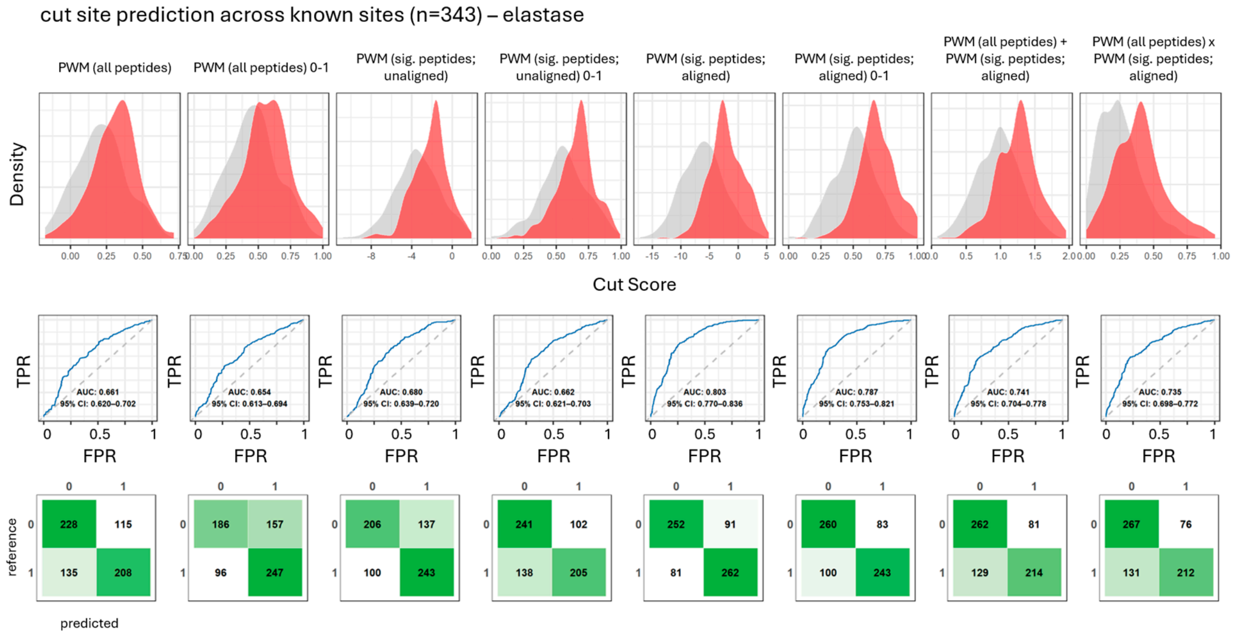

Figure S10: Classification performance of phage-derived cut scores for elastase. Performance was evaluated in a balanced dataset with a 1:1 ratio of MEROPs to random cut-sites.

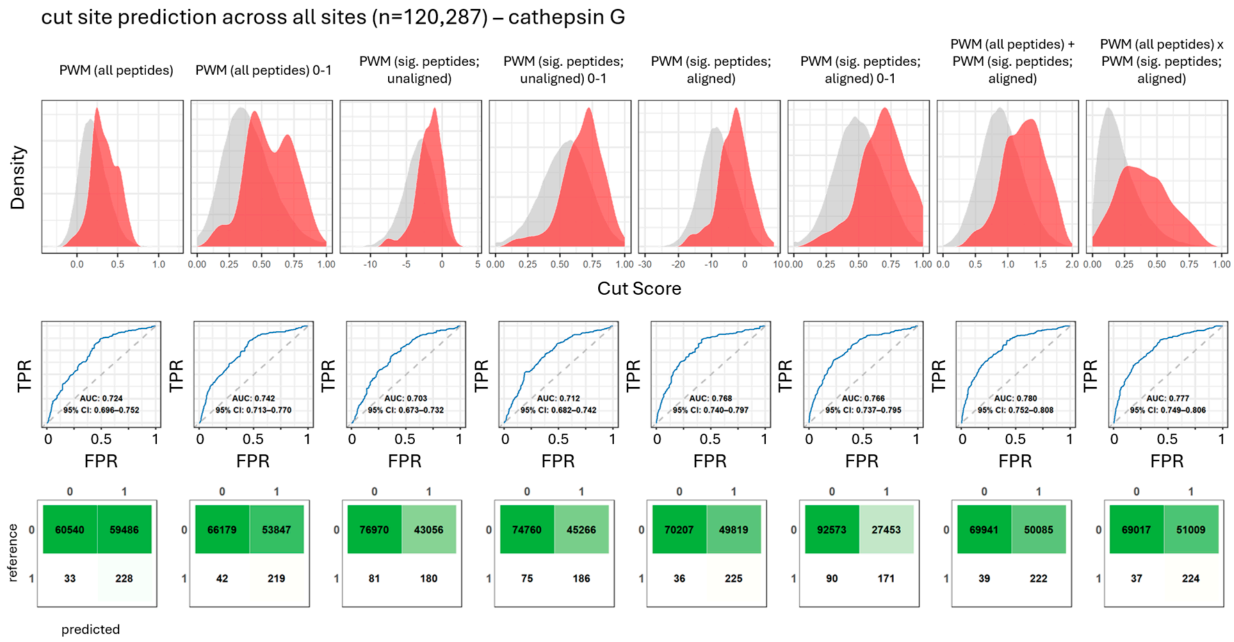

Figure S11: Classification performance of phage-derived cut scores for cathepsin G. Performance was tested across all possible cut-sites for the substrates.

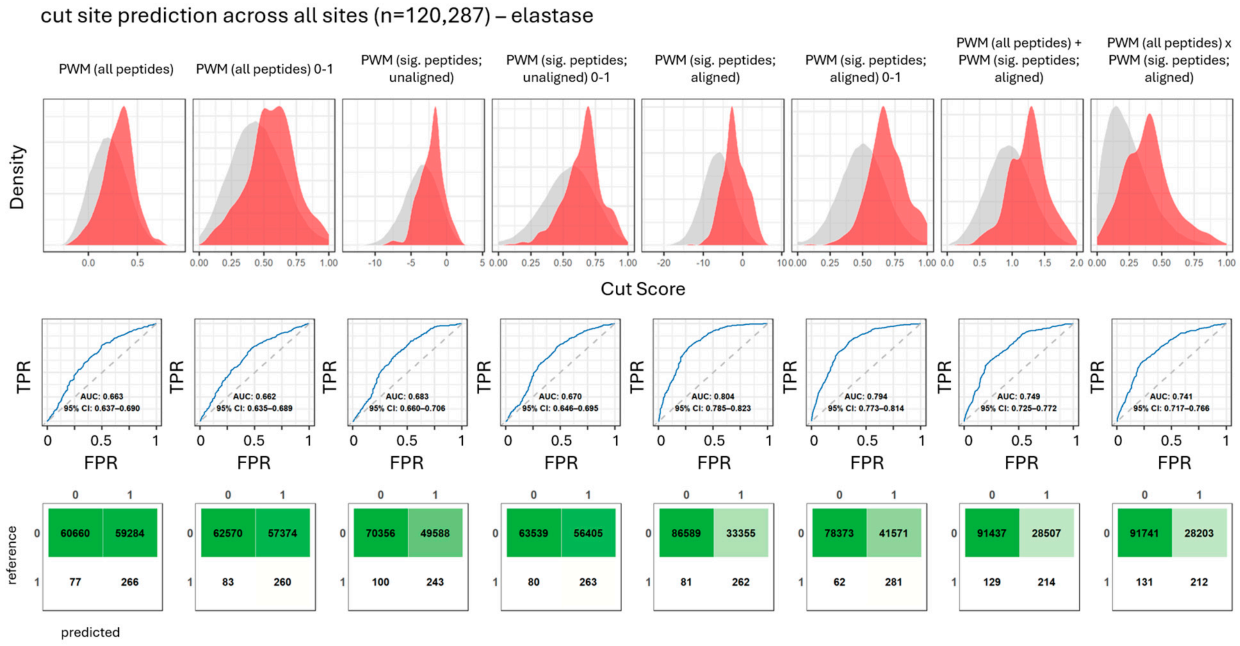

Figure S12: Classification performance of phage-derived cut scores for elastase. Performance was tested across all possible cut-sites for the substrates.

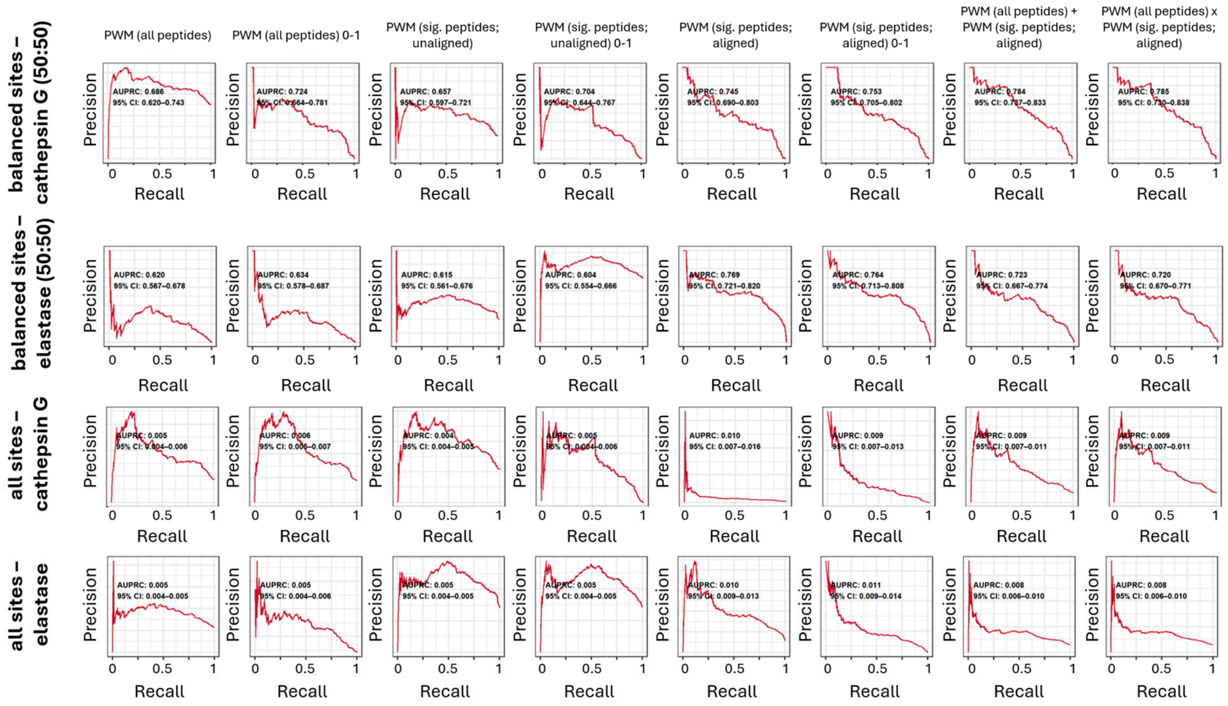

Figure S13: Precision-recall curve performance of phage-derived cut scores for cathepsin G or elastase. Performance was tested either for a balanced dataset with a 1:1 ratio of MEROPs to random cut-sites, or across all possible cut-sites for the substrates.

Table S3: Performance of phage-based scoring for cathepsin G or elastase on either a balanced (1:1 ratio) or full dataset.

Metric	PWM (all peptides)	PWM (all peptides) 0-1	PWM (sig. peptides; unaligned)	PWM (sig. peptides; unaligned) 0-1	PWM (sig. peptides; aligned)	PWM (sig. peptides; aligned) 0-1	PWM (all peptides) + PWM (sig. peptides; aligned)	PWM (all peptides) x PWM (sig. peptides; aligned)
**Balanced Dataset**								
**Cathepsin G**								
AUC	0.726 (95% CI: 0.683-0.769)	0.751 (95% CI: 0.709-0.792)	0.706 (95% CI: 0.662-0.751)	0.729 (95% CI: 0.686-0.772)	0.753 (95% CI: 0.712-0.795)	0.756 (95% CI: 0.714-0.797)	0.783 (95% CI: 0.744-0.822)	0.782 (95% CI: 0.743-0.821)
AUPRC	0.686 (95% CI: 0.620-0.743)	0.724 (95% CI: 0.664-0.781)	0.657 (95% CI: 0.597-0.721)	0.704 (95% CI: 0.644-0.767)	0.745 (95% CI: 0.690-0.803)	0.753 (95% CI: 0.705-0.802)	0.784 (95% CI: 0.737-0.833)	0.785 (95% CI: 0.730-0.838)
Sensitivity	0.874 (95% CI: 0.828-0.909)	0.854 (95% CI: 0.806-0.892)	0.705 (95% CI: 0.647-0.757)	0.529 (95% CI: 0.468-0.588)	0.874 (95% CI: 0.828-0.909)	0.805 (95% CI: 0.752-0.848)	0.851 (95% CI: 0.802-0.889)	0.858 (95% CI: 0.811-0.895)
Specificity	0.494 (95% CI: 0.434-0.555)	0.544 (95% CI: 0.483-0.603)	0.644 (95% CI: 0.584-0.699)	0.858 (95% CI: 0.811-0.895)	0.552 (95% CI: 0.491-0.611)	0.598 (95% CI: 0.537-0.655)	0.586 (95% CI: 0.526-0.644)	0.586 (95% CI: 0.526-0.644)
PPV	0.633 (95% CI: 0.582-0.681)	0.652 (95% CI: 0.600-0.701)	0.664 (95% CI: 0.607-0.717)	0.789 (95% CI: 0.722-0.843)	0.661 (95% CI: 0.609-0.709)	0.667 (95% CI: 0.613-0.716)	0.673 (95% CI: 0.620-0.721)	0.675 (95% CI: 0.623-0.723)
NPV	0.796 (95% CI: 0.728-0.851)	0.789 (95% CI: 0.724-0.842)	0.686 (95% CI: 0.625-0.741)	0.646 (95% CI: 0.594-0.694)	0.814 (95% CI: 0.750-0.864)	0.754 (95% CI: 0.691-0.807)	0.797 (95% CI: 0.734-0.848)	0.805 (95% CI: 0.743-0.855)
MCC	0.398	0.419	0.349	0.41	0.449	0.411	0.453	0.462
**Elastase**								
AUC	0.661 (95% CI: 0.620-0.702)	0.654 (95% CI: 0.613-0.694)	0.680 (95% CI: 0.639-0.720)	0.662 (95% CI: 0.621-0.703)	0.803 (95% CI: 0.770-0.836)	0.787 (95% CI: 0.753-0.821)	0.741 (95% CI: 0.704-0.778)	0.735 (95% CI: 0.698-0.772)

AUPRC	0.620 (95% CI: 0.567-0.678)	0.634 (95% CI: 0.578-0.687)	0.615 (95% CI: 0.561-0.676)	0.604 (95% CI: 0.554-0.666)	0.769 (95% CI: 0.721-0.820)	0.764 (95% CI: 0.713-0.808)	0.723 (95% CI: 0.667-0.774)	0.720 (95% CI: 0.670-0.771)
Sensitivity	0.606 (95% CI: 0.554-0.657)	0.720 (95% CI: 0.670-0.765)	0.708 (95% CI: 0.658-0.754)	0.598 (95% CI: 0.545-0.648)	0.764 (95% CI: 0.716-0.806)	0.708 (95% CI: 0.658-0.754)	0.624 (95% CI: 0.572-0.674)	0.618 (95% CI: 0.566-0.668)
Specificity	0.665 (95% CI: 0.613-0.713)	0.542 (95% CI: 0.489-0.594)	0.601 (95% CI: 0.548-0.651)	0.703 (95% CI: 0.652-0.749)	0.735 (95% CI: 0.686-0.779)	0.758 (95% CI: 0.710-0.800)	0.764 (95% CI: 0.716-0.806)	0.778 (95% CI: 0.732-0.819)
PPV	0.644 (95% CI: 0.590-0.694)	0.611 (95% CI: 0.563-0.658)	0.639 (95% CI: 0.590-0.686)	0.668 (95% CI: 0.613-0.718)	0.742 (95% CI: 0.694-0.785)	0.745 (95% CI: 0.695-0.790)	0.725 (95% CI: 0.672-0.773)	0.736 (95% CI: 0.682-0.784)
NPV	0.628 (95% CI: 0.577-0.676)	0.660 (95% CI: 0.602-0.712)	0.673 (95% CI: 0.619-0.723)	0.636 (95% CI: 0.586-0.683)	0.757 (95% CI: 0.708-0.800)	0.722 (95% CI: 0.674-0.766)	0.670 (95% CI: 0.622-0.715)	0.671 (95% CI: 0.623-0.715)
MCC	0.272	0.267	0.311	0.302	0.499	0.467	0.392	0.402
**Full Dataset**								
**Cathepsin G**								
AUC	0.724 (95% CI: 0.696-0.752)	0.742 (95% CI: 0.713-0.770)	0.703 (95% CI: 0.673-0.732)	0.712 (95% CI: 0.682-0.742)	0.768 (95% CI: 0.740-0.797)	0.766 (95% CI: 0.737-0.795)	0.780 (95% CI: 0.752-0.808)	0.777 (95% CI: 0.749-0.806)
AUPRC	0.005 (95% CI: 0.004-0.006)	0.006 (95% CI: 0.005-0.007)	0.004 (95% CI: 0.004-0.005)	0.005 (95% CI: 0.004-0.006)	0.010 (95% CI: 0.007-0.016)	0.009 (95% CI: 0.007-0.013)	0.009 (95% CI: 0.007-0.011)	0.009 (95% CI: 0.007-0.011)
Sensitivity	0.874 (95% CI: 0.828-0.909)	0.839 (95% CI: 0.790-0.879)	0.690 (95% CI: 0.631-0.743)	0.713 (95% CI: 0.655-0.764)	0.862 (95% CI: 0.815-0.899)	0.655 (95% CI: 0.596-0.710)	0.851 (95% CI: 0.802-0.889)	0.858 (95% CI: 0.811-0.895)
Specificity	0.504 (95% CI: 0.502-0.507)	0.551 (95% CI: 0.549-0.554)	0.641 (95% CI: 0.639-0.644)	0.623 (95% CI: 0.620-0.626)	0.585 (95% CI: 0.582-0.588)	0.771 (95% CI: 0.769-0.774)	0.583 (95% CI: 0.580-0.586)	0.575 (95% CI: 0.572-0.578)
PPV	0.004 (95% CI: 0.003-0.004)	0.004 (95% CI: 0.004-0.005)	0.004 (95% CI: 0.004-0.005)	0.004 (95% CI: 0.004-0.005)	0.004 (95% CI: 0.004-0.005)	0.006 (95% CI: 0.005-0.007)	0.004 (95% CI: 0.004-0.005)	0.004 (95% CI: 0.004-0.005)
NPV	0.999 (95% CI: 0.999-1.000)	0.999 (95% CI: 0.999-1.000)	0.999 (95% CI: 0.999-0.999)	0.999 (95% CI: 0.999-0.999)	0.999 (95% CI: 0.999-1.000)	0.999 (95% CI: 0.999-0.999)	0.999 (95% CI: 0.999-1.000)	0.999 (95% CI: 0.999-1.000)
MCC	0.035	0.037	0.032	0.032	0.042	0.047	0.041	0.041
**Elastase**								
AUC	0.663 (95% CI: 0.637-0.690)	0.662 (95% CI: 0.635-0.689)	0.683 (95% CI: 0.660-0.706)	0.670 (95% CI: 0.646-0.695)	0.804 (95% CI: 0.785-0.823)	0.794 (95% CI: 0.773-0.814)	0.749 (95% CI: 0.725-0.772)	0.741 (95% CI: 0.717-0.766)
AUPRC	0.005 (95% CI: 0.004-0.005)	0.005 (95% CI: 0.004-0.006)	0.005 (95% CI: 0.004-0.005)	0.005 (95% CI: 0.004-0.005)	0.010 (95% CI: 0.008-0.013)	0.011 (95% CI: 0.009-0.014)	0.008 (95% CI: 0.006-0.010)	0.008 (95% CI: 0.006-0.010)
Sensitivity	0.776 (95% CI: 0.728-0.816)	0.758 (95% CI: 0.710-0.800)	0.708 (95% CI: 0.658-0.754)	0.767 (95% CI: 0.719-0.808)	0.764 (95% CI: 0.716-0.806)	0.819 (95% CI: 0.775-0.856)	0.624 (95% CI: 0.572-0.674)	0.618 (95% CI: 0.566-0.668)
Specificity	0.506 (95% CI: 0.503-0.509)	0.522 (95% CI: 0.519-0.524)	0.587 (95% CI: 0.584-0.589)	0.530 (95% CI: 0.527-0.533)	0.722 (95% CI: 0.719-0.724)	0.653 (95% CI: 0.651-0.656)	0.762 (95% CI: 0.760-0.765)	0.765 (95% CI: 0.762-0.767)
PPV	0.004 (95% CI: 0.004-0.005)	0.005 (95% CI: 0.004-0.005)	0.005 (95% CI: 0.004-0.006)	0.005 (95% CI: 0.004-0.005)	0.008 (95% CI: 0.007-0.009)	0.007 (95% CI: 0.006-0.008)	0.007 (95% CI: 0.007-0.009)	0.007 (95% CI: 0.007-0.009)
NPV	0.999 (95% CI: 0.998-0.999)	0.999 (95% CI: 0.998-0.999)	0.999 (95% CI: 0.998-0.999)	0.999 (95% CI: 0.998-0.999)	0.999 (95% CI: 0.999-0.999)	0.999 (95% CI: 0.999-0.999)	0.999 (95% CI: 0.998-0.999)	0.999 (95% CI: 0.998-0.999)
MCC	0.03	0.03	0.032	0.032	0.058	0.053	0.048	0.048

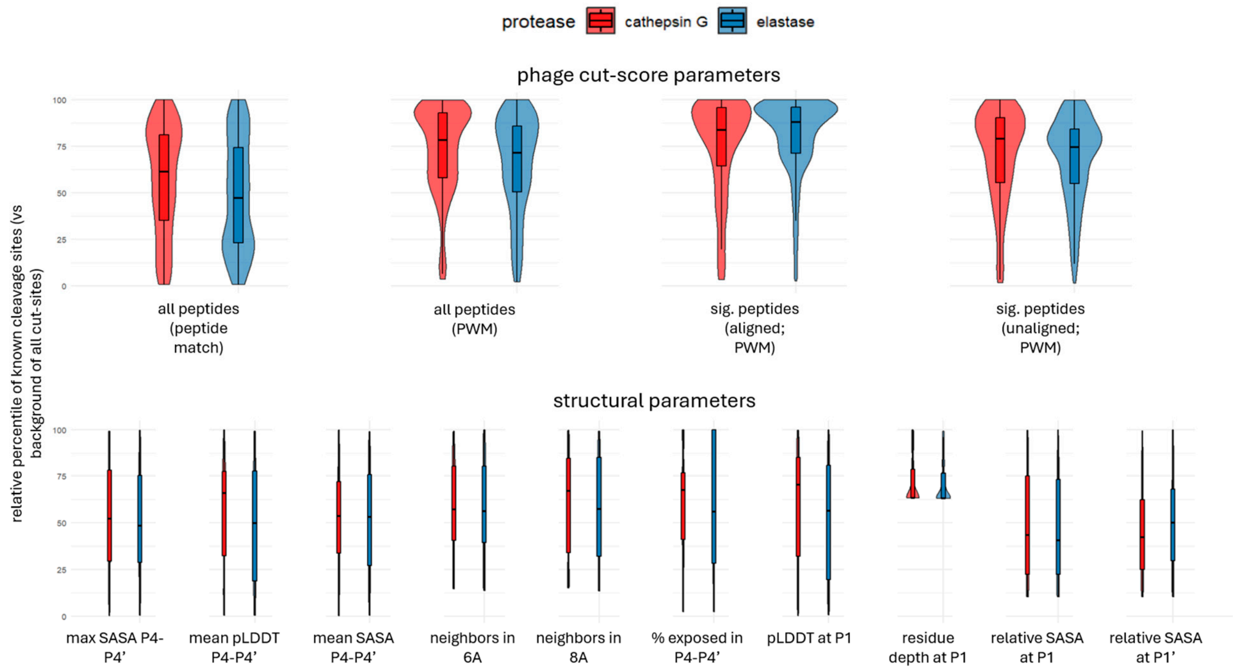

Figure S14: Violin plot of percentiles averaged across all known cleavage sites, relative to background cut-sites, for structural and phage-derived parameters

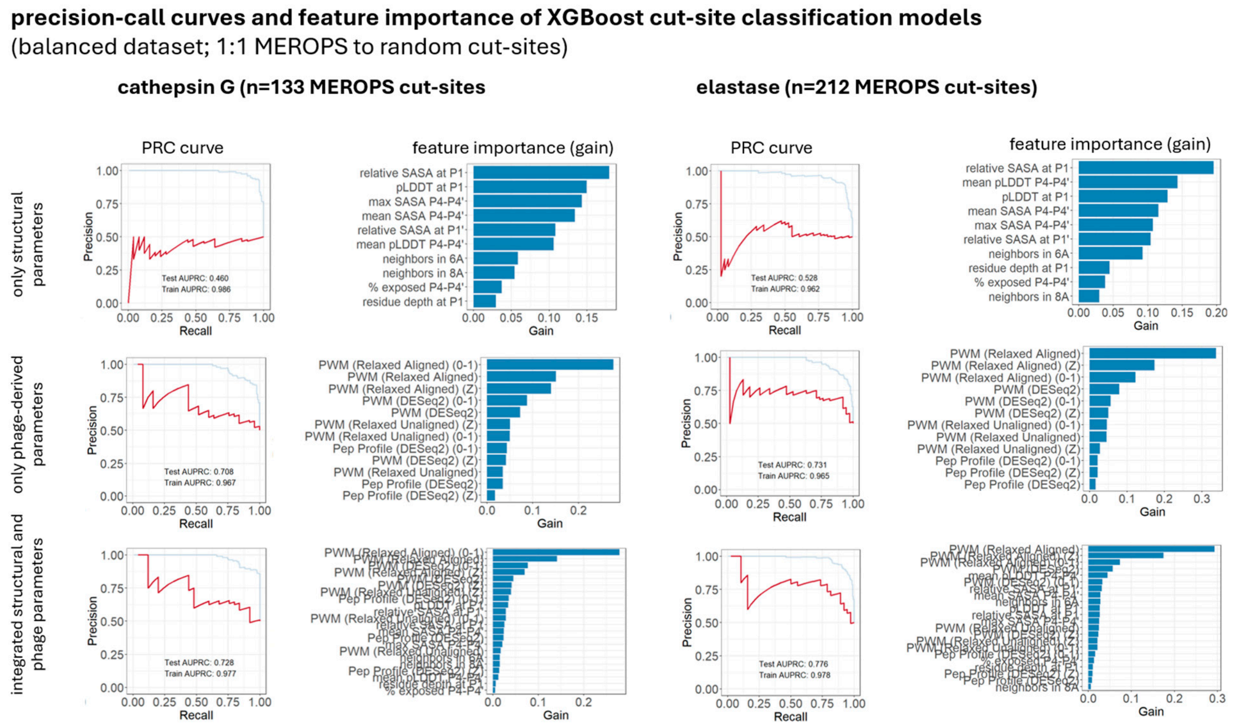

Figure S15: Feature importance of XGBoost classification models for cathepsin G and elastase trained on a balanced dataset with a 1:1 ratio of MEROPs to random cut-sites.

XGBoost cut-site classification models (full dataset; across all cut-sites)

cathepsin G (n=133 MEROPS cut-sites; 132 filtered out due to high cleavage site or substrate similarity)

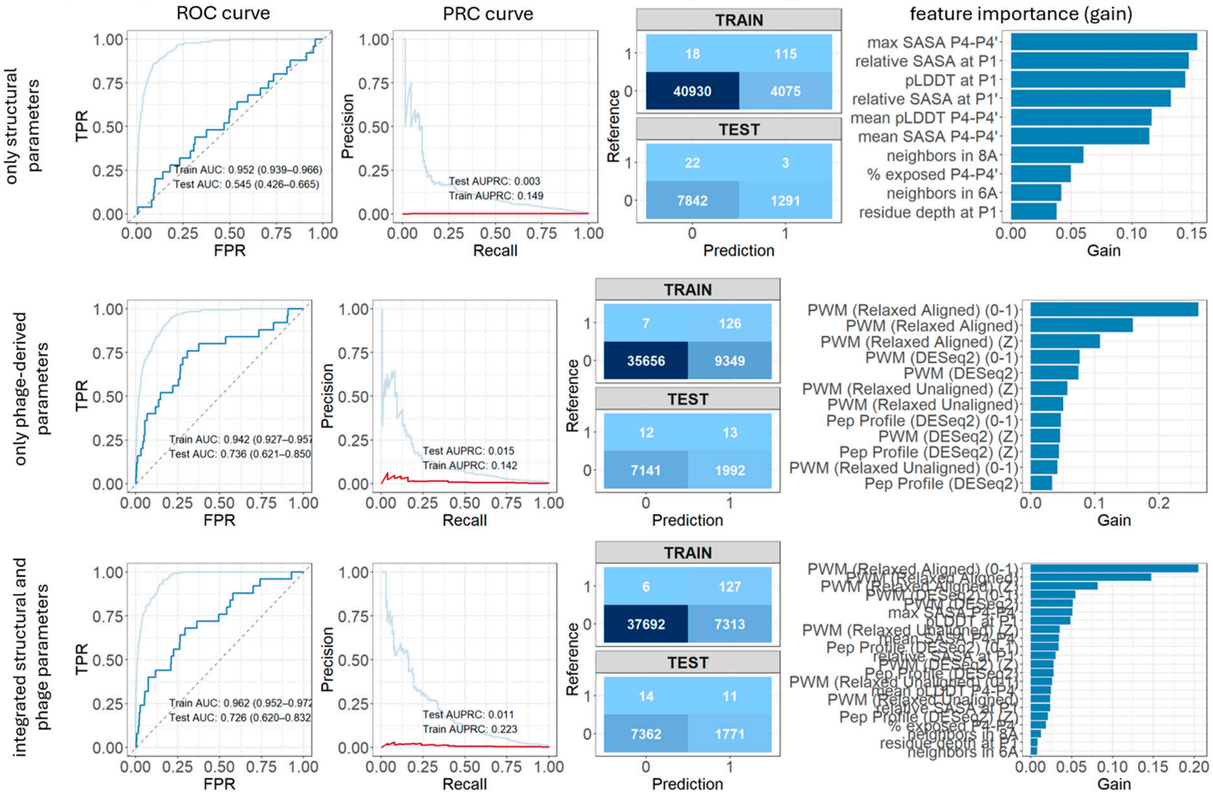

Figure S16: XGBoost classification models for cathepsin G trained on the full dataset, with predictions across allpossible cut-sites for the substrates.

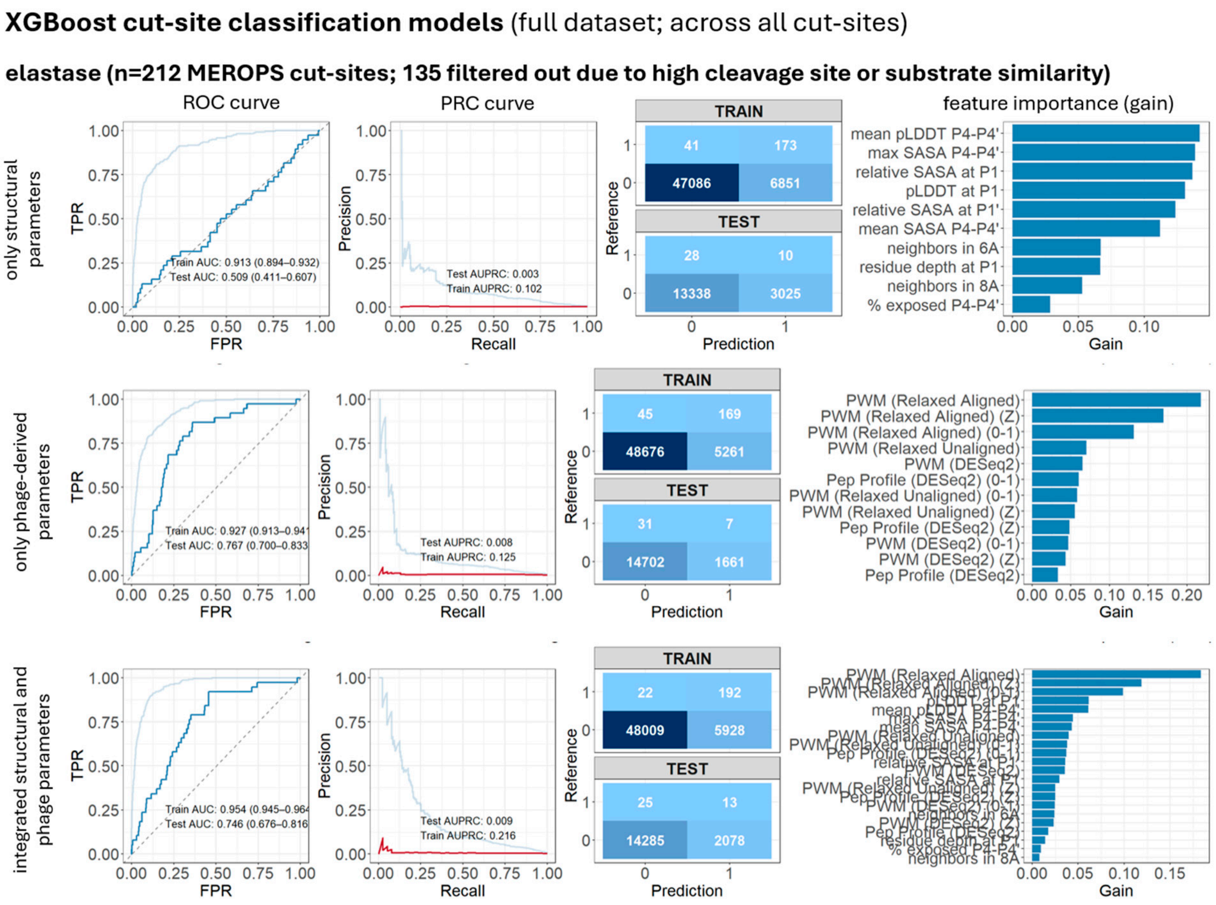

Figure S17: XGBoost classification models for elastase trained on the full dataset, with predictions across all possible cut-sites for the substrates.

Table S4: Performance of XGBoost classification models for cathepsin G and elastase trained on a balanced dataset with a 1:1 ratio of MEROPs to random cut-sites.*

	**cathepsin G - structural**	**cathepsin G - phage**	**cathepsin G – phage and structural**	**elastase - structural**	**elastase - phage**	**elastase - phage and structural**
Train AUC	0.985 (0.97-1.00)	0.966 (0.95-0.98)	0.977 (0.96-0.99)	0.962 (0.95-0.98)	0.962 (0.95-0.98)	0.976 (0.97-0.99)
Test AUC	0.600 (0.44-0.76); CV 0.531 (0.42-0.64)	0.702 (0.56-0.85); CV 0.791 (0.74-0.84)	0.710 (0.56-0.86); CV 0.803 (0.74-0.86)	0.533 (0.40-0.67); CV 0.535 (0.45-0.62)	0.775 (0.67-0.88); CV 0.751 (0.65-0.85)	0.806 (0.70-0.91); CV 0.763 (0.67-0.86)
Train AUPRC	0.986	0.967	0.977	0.962	0.965	0.978
Test AUPRC	0.460; CV 0.605 (0.57-0.64)	0.708; CV 0.813 (0.74-0.89)	0.728; CV 0.828 (0.77-0.89)	0.528; CV 0.566 (0.52-0.62)	0.731; CV 0.736 (0.61-0.87)	0.776; CV 0.752 (0.63-0.88)
Test Sensitivity	0.640; CV 0.523 (0.43-0.61)	0.800; CV 0.720 (0.65-0.79)	0.760; CV 0.746 (0.65-0.84)	0.632; CV 0.563 (0.41-0.71)	0.816; CV 0.680 (0.58-0.78)	0.789; CV 0.730 (0.60-0.86)
Test Specificity	0.200; CV 0.563 (0.50-0.63)	0.520; CV 0.734 (0.68-0.78)	0.600; CV 0.724 (0.60-0.85)	0.395; CV 0.547 (0.40-0.69)	0.605; CV 0.717 (0.54-0.89)	0.737; CV 0.687 (0.54-0.84)
Test PPV	0.444; CV 0.544 (0.48-0.61)	0.625; CV 0.731 (0.69-0.77)	0.655; CV 0.735 (0.66-0.81)	0.511; CV 0.554 (0.49-0.61)	0.674; CV 0.717 (0.60-0.84)	0.750; CV 0.708 (0.63-0.79)

Test NPV	0.357; CV 0.543 (0.48-0.60)	0.722; CV 0.725 (0.68-0.78)	0.714; CV 0.743 (0.68-0.81)	0.517; CV 0.557 (0.50-0.62)	0.767; CV 0.691 (0.62-0.76)	0.778; CV 0.725 (0.66-0.79)
Test MCC	-0.178; CV 0.087 (-0.04-0.21)	0.333; CV 0.455 (0.38-0.53)	0.365; CV 0.473 (0.36-0.59)	0.027; CV 0.110 (-0.01-0.23)	0.431; CV 0.402 (0.23-0.57)	0.527; CV 0.425 (0.33-0.52)
Test F1	0.525; CV 0.533 (0.46-0.61)	0.702; CV 0.724 (0.68-0.77)	0.704; CV 0.737 (0.68-0.79)	0.565; CV 0.553 (0.46-0.65)	0.738; CV 0.694 (0.62-0.77)	0.769; CV 0.713 (0.66-0.77)

*CV: 5-fold cross-validation

Table S5: Performance of XGBoost classification models for cathepsin G and elastase trained on the full dataset, with predictions across all possible cut-sites for the substrates.*

	**cathepsin G - structural**	**cathepsin G - phage**	**cathepsin G – phage and structural**	**elastase - structural**	**elastase - phage**	**elastase - phage and structural**
Train AUC	0.952 (0.94-0.97)	0.942 (0.93-0.96)	0.962 (0.95-0.97)	0.913 (0.89-0.93)	0.927 (0.91-0.94)	0.954 (0.94-0.96)
Test AUC	0.545 (0.43-0.66); CV 0.553 (0.52-0.59)	0.736 (0.62-0.85); CV 0.792 (0.74-0.85)	0.726 (0.62-0.83); CV 0.799 (0.75-0.85)	0.509 (0.41-0.61); CV 0.558 (0.50-0.61)	0.767 (0.70-0.83); CV 0.769 (0.74-0.80)	0.746 (0.68-0.82); CV 0.777 (0.75-0.81)
Train AUPRC	0.149	0.142	0.223	0.102	0.125	0.216
Test AUPRC	0.003; CV 0.004 (0.00-0.00)	0.015; CV 0.017 (0.01-0.02)	0.011; CV 0.027 (0.01-0.04)	0.003; CV 0.006 (0.00-0.01)	0.008; CV 0.011 (0.01-0.02)	0.009; CV 0.014 (0.01-0.02)
Test Sensitivity	0.120; CV 0.183 (0.10-0.26)	0.520; CV 0.657 (0.51-0.80)	0.440; CV 0.595 (0.48-0.71)	0.263; CV 0.278 (0.13-0.42)	0.184; CV 0.476 (0.36-0.59)	0.342; CV 0.420 (0.33-0.51)
Test Specificity	0.859; CV 0.858 (0.81-0.90)	0.782; CV 0.825 (0.78-0.87)	0.806; CV 0.856 (0.82-0.89)	0.815; CV 0.816 (0.72-0.92)	0.898; CV 0.829 (0.80-0.86)	0.873; CV 0.868 (0.84-0.90)
Test PPV	0.002; CV 0.004 (0.00-0.01)	0.006; CV 0.011 (0.01-0.01)	0.006; CV 0.012 (0.01-0.01)	0.003; CV 0.006 (0.00-0.01)	0.004; CV 0.010 (0.00-0.02)	0.006; CV 0.012 (0.00-0.02)
Test NPV	0.997; CV 0.997 (1.00-1.00)	0.998; CV 0.999 (1.00-1.00)	0.998; CV 0.999 (1.00-1.00)	0.998; CV 0.997 (1.00-1.00)	0.998; CV 0.998 (1.00-1.00)	0.998; CV 0.998 (1.00-1.00)
Test MCC	-0.003; CV 0.006 (0.00-0.01)	0.038; CV 0.068 (0.06-0.08)	0.032; CV 0.069 (0.06-0.08)	0.010; CV 0.015 (0.00-0.03)	0.013; CV 0.048 (0.03-0.06)	0.031; CV 0.051 (0.03-0.08)
Test F1	0.005; CV 0.007 (0.01-0.01)	0.013; CV 0.022 (0.02-0.03)	0.012; CV 0.024 (0.02-0.03)	0.007; CV 0.011 (0.00-0.02)	0.008; CV 0.019 (0.01-0.03)	0.012; CV 0.023 (0.01-0.04)

*CV: 5-fold cross-validation
